# Comparative analysis of three BMI cutoffs for five metabolic abnormalities in the population of Western Guangdong, China

**DOI:** 10.3389/fnut.2025.1732345

**Published:** 2025-12-03

**Authors:** Dengwen Pang, Meizhu Chen, Chengwen Xiao, Ruijian Zhuang, Chengyu Kong, Mengyuan Xiao

**Affiliations:** 1 Department of Cardiovascular Medicine Center, Affiliated Hospital of Guangdong Medical University, Zhanjiang, China; 2 Department of Assets and Equipment Management, Guangdong Medical University, Zhanjiang, Guangdong, China; 3 Dongxin Street Community Health Service Center, Zhanjiang, Guangdong, China; 4 The First Clinical College, Guangdong Medical University, Zhanjiang, Guangdong, China

**Keywords:** body mass index, metabolic risk, “5 highs”, obesity, Western Guangdong population

## Abstract

**Background and objective:**

Obesity is a core risk factor for metabolic diseases, and body mass index (BMI) serves as a key tool for assessing obesity. Current internationally adopted BMI standards (such as the WHO universal standard and the Asia-Pacific standard) differ from the Chinese standard developed based on data from the Chinese population. Given the unique genetic background and lifestyle of the population in Western Guangdong, China, their spectrum of metabolic diseases may exhibit distinct characteristics. This study aims to systematically compare the predictive capabilities of the three commonly used BMI standards for the “five highs” (hypertension, dyslipidemia, diabetes, hyperuricemia, and hyperhomocysteinemia) and their clustering risk in this population, providing evidence for localized precision prevention and control.

**Methods:**

This cross-sectional study included 5,667 permanent residents in Western Guangdong who completed health examinations between 2023 and 2024. Participants were grouped according to three BMI standards: Chinese (overweight ≥24 kg/m^2^, obese ≥28 kg/m^2^); WHO General (overweight ≥25 kg/m^2^, obese ≥30 kg/m^2^); WHO Asia-Pacific (overweight ≥23 kg/m^2^, obese ≥25 kg/m^2^). Multivariate logistic regression and restricted cubic spline (RCS) analyses examined the association between BMI and the risk of “five highs” (hyperglycemia, hyperlipidemia, hypertension, hyperuricemia and hyperhomocysteinemia) and their clustering. Subgroup analyses by age and sex were conducted.

**Results:**

Individuals classified as overweight or obese according to Chinese criteria showed the strongest risk gradient for clustering two or more metabolic risks, with overweight and obesity increasing the risk of the five high by 79 and 140%, respectively. RCS analysis revealed a nonlinear positive correlation between BMI and the risk of clustering the “five highs,” with the most significant change in risk slope occurring near the cut-off points defined by the Chinese standard (24 and 28 kg/m^2^). Subgroup analysis further indicated that males and younger individuals exhibited heightened sensitivity to metabolic risks associated with increasing BMI.

**Conclusion:**

Among the population in Western Guangdong, China, the Chinese BMI standard outperformed both the WHO universal and Asia-Pacific standards in identifying “five highs” and their clustered risks, demonstrating greater sensitivity in screening high-risk individuals. It is recommended as the preferred tool for primary prevention of metabolic diseases and public health strategy development in this region.

## Introduction

Obesity is one of the core risk factors for metabolic diseases. As the primary indicator for assessing obesity severity (1), the body mass index (BMI) classification thresholds directly impact the accuracy of disease risk evaluation. Currently, internationally adopted BMI standards primarily include the World Health Organization (WHO) universal standards (overweight: BMI ≥ 25 kg/m^2^; obese: BMI ≥ 30 kg/m^2^) (2); the WHO Asia-Pacific standards (overweight: BMI ≥ 23 kg/m^2^; obese: BMI ≥ 25 kg/m^2^) (3) and the Chinese standards (overweight: BMI ≥ 24 kg/m^2^; obese: BMI ≥ 28 kg/m^2^) (4, 5). Asian populations require stricter thresholds due to unique body fat distribution and metabolic risk profiles. The Chinese criteria, developed based on domestic epidemiological data, better align with the physiological characteristics of the Chinese population (6).

The clustering phenomenon of metabolic risk factors—the “five highs” (hypertension, dyslipidemia, diabetes, hyperuricemia, and hyperhomocysteinemia) represents a cluster of closely interrelated metabolic risk factors. Their detrimental effects on health are comprehensive and multisystemic, serving as core drivers of atherosclerotic cardiovascular disease (ASCVD), major causes of end-stage renal disease, and contributors to cerebrovascular disorders and cognitive impairment. Additionally, the “five highs” can lead to other severe complications such as retinopathy, fatty liver disease, osteoporosis, and increased risk of certain cancers (7–16). Metabolically, significant synergistic effects exist among these five conditions: obesity and insulin resistance simultaneously promote hypertension, dyslipidemia (17–19), and hyperuricemia; hyperuricemia exacerbates metabolic syndrome and type 2 diabetes (20). Hyperhomocysteinemia is not only an independent risk factor for cardiovascular and cerebrovascular diseases but also exhibits well-documented synergistic interactions with other metabolic abnormalities. For instance, hyperhomocysteinemia can promote endothelial dysfunction and oxidative stress, which may amplify the atherogenic effects of hypertension and dyslipidemia. Hyperhomocysteinemia impairs glucose control by promoting insulin resistance (21). This synergistic effect creates a more detrimental cardiometabolic risk profile than any single factor alone.

The Western Guangdong region, as a representative population in southern China, possesses unique genetic backgrounds, tropical monsoon and marine climatic conditions, and a lifestyle characterized by high seafood consumption. This region has a high prevalence of seafood consumption, which may influence homocysteine and uric acid levels. Additionally, genetic studies have suggested a higher frequency of certain metabolic-related gene variants in southern Chinese populations compared to northern groups (22, 23). Consequently, its spectrum of metabolic diseases may differ from the national average. However, systematic research remains lacking on which BMI standard most effectively identifies the risk of “five highs” clustering in this population. Different BMI thresholds could lead to significant variations in the target populations for prevention strategies and the allocation of public health resources.

Thus, this study aims to systematically compare the predictive capabilities and overall correlation strength of the Chinese standard, WHO universal standard, and WHO Asia-Pacific standard for the risk of developing the “five highs” (hypertension, dyslipidemia, diabetes, hyperuricemia, and hyperhomocysteinemia) based on epidemiological data from the population in Western Guangdong. This will provide scientific evidence for formulating precise primary prevention strategies for obesity and related metabolic diseases in this region.

## Materials and methods

### Study design and population

**Figure 1:**
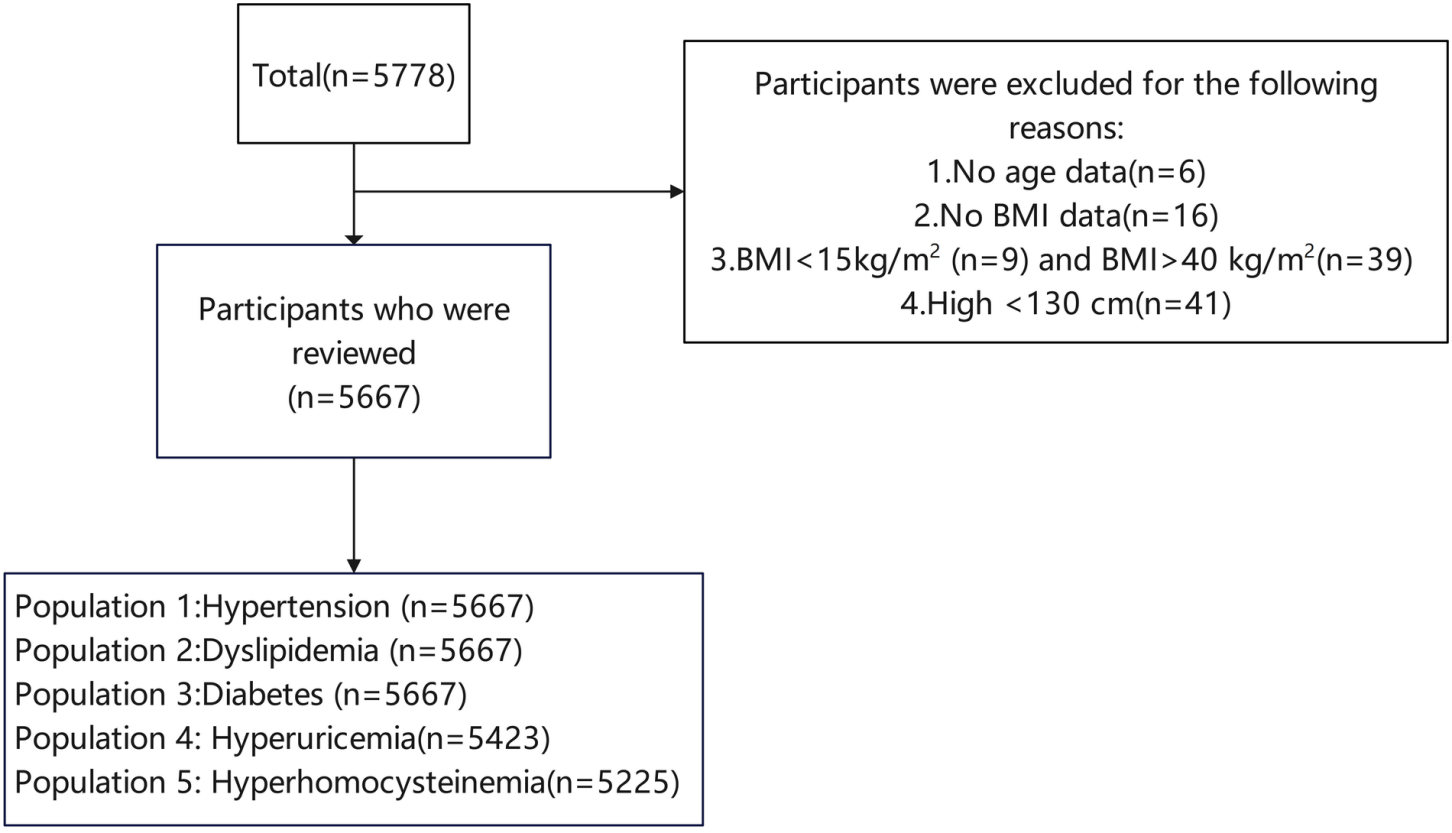
The population selection process.

This study included permanent residents who completed basic public health physical examinations at the Zhanjiang Municipal Health Service Center during 2023–2024. Local household registration, voluntary participation with signed informed consent, and available BMI measurement data were included. BMI outside the acceptable range (15–40 kg/m^2^) (24) or missing key covariates (age, gender) were excluded. Excluding individuals with extreme BMI is to reduce the potential interference of comorbidities related to severe malnutrition and pathological obesity on the research results. The screening process for the study population is illustrated in Figure 1.

### Ethics statement

This study was approved by the Ethics Committee of the Affiliated Hospital of Guangdong Medical University, Guangdong Province, China (Approval Number: PJKT2023-162). All subjects included in the study signed informed consent forms.

### BMI measurement

This study utilized height and weight data measured by investigators during physical examinations, excluding extreme measurements (height range: 130–200 cm; weight range: 30–150 kg). All anthropometric measurements (height, weight, waist circumference) were conducted by a team of trained and certified health professionals. To ensure consistency, a standardization session was held prior to the commencement of the study, where all observers measured the same set of volunteers. The technical error of measurement (TEM) and intra-class correlation coefficients (ICC) were calculated, demonstrating excellent agreement (ICC > 0.95). Periodic re-calibration sessions were conducted throughout the data collection period. BMI was calculated as weight (kg) divided by height squared (m^2^). Initially, BMI was analyzed as a continuous variable. Subsequently, BMI was categorized according to threshold standards established by the WHO and the Chinese Working Group, with the WHO standards further divided into general criteria and Asia-Pacific criteria. Researchers employed three classification approaches:

1. Chinese criteria: Normal weight (18.5 kg/m^2^ ≤ BMI < 24 kg/m^2^), overweight (24 kg/m^2^ ≤ BMI < 28 kg/m^2^), and obese (BMI ≥ 28 kg/m^2^) (2);
2. WHO general criteria: Normal weight (18.5 kg/m^2^ ≤ BMI < 25 kg/m^2^), overweight (25 kg/m^2^ ≤ BMI < 30 kg/m^2^), and obesity (BMI ≥ 30 kg/m^2^) (3);
3. WHO Asia Pacific criteria: Normal weight (18.5 kg/m^2^ ≤ BMI < 23 kg/m^2^), overweight (23 kg/m^2^ ≤ BMI < 25 kg/m^2^), and obese (BMI ≥ 25 kg/m^2^) (4).

### Covariates of interest

Trained investigators collected participants’ baseline sociodemographic data using a structured questionnaire, including age, gender, marital status, past or current smoking status, alcohol consumption in the past year, history of hypertension and diabetes, and allergy history. Marital status options included married with spouse living together, unmarried, and other. The “married with spouse living together” category was classified as “married,” while the remaining options were grouped under “other marital status.” Blood pressure measurement: Following the standards established in the 2018 Revised Chinese Hypertension Prevention and Treatment Guidelines, blood pressure was measured twice using an Omron HEM-7430 electronic blood pressure monitor after 5 min of seated rest. The average of the two readings is recorded. If a difference exceeding 5 mmHg is detected, measurements are repeated, and the average of three readings is used. Waist circumference measurement: Measure at the midpoint between the iliac crest and the lower edge of the 12th rib. Ensure the tape measure remains horizontal throughout, fitting snugly without compressing the skin. Waist circumference is also recorded in centimeters, accurate to the nearest centimeter.

### Laboratory measurement indicators

All laboratory analyses were performed in a certified central laboratory. All instruments (e.g., automated biochemical analyzers) were calibrated daily using manufacturer-provided calibration kits and quality control materials. We adhered to a strict internal quality control (IQC) protocol, running low and high concentration control samples with each batch of patient samples. After fasting blood collection, all blood samples were allowed to stand for 30 min. Serum/plasma was separated by centrifugation at 3,000 rpm for 10 min and stored at −80 °C to avoid repeated freeze–thaw cycles. Serum total cholesterol (TC), triglycerides (TG), high-density lipoprotein cholesterol (HDL-C), and low-density lipoprotein cholesterol (LDL-C) were measured using immunoturbidimetry (transmission method). Glucose levels were measured using the glucose oxidase-peroxidase (GOD-POD) method. Glycated hemoglobin was determined by immunoturbidimetry. Serum uric acid was detected via the uricase-peroxidase method. Homocysteine levels were measured using the enzyme-cyclic method or high-performance liquid chromatography (HPLC).

### Primary endpoints

This study included participants with hypertension, dyslipidemia, diabetes, hyperuricemia, and hyperhomocysteinemia, diagnosed according to the following criteria:

1. Hypertension: According to the 2018 Revised Chinese Hypertension Prevention and Treatment Guidelines, meeting any one of the following: (1) Mean systolic blood pressure ≥140 mmHg and/or mean diastolic blood pressure ≥90 mmHg; (2) Self-reported hypertension; (3) Self-reported past or current use of antihypertensive medication (25).
2. Dyslipidemia: Total cholesterol (TC) ≥ 6.2 mmol/L, or low-density lipoprotein cholesterol (LDL-C) ≥ 4.1 mmol/L, or triglycerides (TG) ≥ 2.3 mmol/L, or high-density lipoprotein cholesterol (HDL-C) < 1.0 mmol/L. (26)
3. Diabetes: (1) Fasting blood glucose ≥7.0 mmol/L or glycated hemoglobin (HbA1c) ≥ 6.5%; (2) Self-reported history of diabetes; (3) Self-reported past or current use of hypoglycemic medications (27).
4. Hypeuricemiar: Serum uric acid > 420 μmol/L (regardless of gender) (28).
5. Hyperhomocysteinemia: Serum homocysteine ≥10 μmol/L. (29).

Multiple metabolic risk is defined as the simultaneous presence of two or more of the above five metabolic diseases in the same individual.

“Two highs”: Simultaneous presence of two metabolic diseases;
“Three highs”: Simultaneous presence of three metabolic diseases;
“Four highs”: Simultaneous presence of four metabolic diseases;
“Five highs”: Simultaneous presence of five metabolic diseases.

### Statistical analysis

Data were categorized into continuous and categorical variables. Continuous variables were further divided into two groups based on their normality of distribution. Normally distributed continuous variables were expressed as mean ± standard deviation and compared between groups using Student’s *t*-test. Concurrently, non-normally distributed variables are presented as median ± interquartile range (IQR) and compared between groups using the Wilcoxon signed-rank test. Categorical variables are expressed as percentages and compared using the chi-square test. For BMI-based participant grouping (Chinese criteria, WHO general criteria, and WHO Asia-Pacific criteria), intergroup differences are assessed for significance using the Kruskal-Wallis test or one-way analysis of variance (ANOVA).

Restricted cubic spline (RCS) terms were employed to assess the nonlinear association between BMI and multiple diseases. Cross-sectional analyses using logistic regression models evaluated the association between weight status and various diseases based on both China criteria and WHO general and Asia-Pacific threshold criteria. In all analyses, following collinearity assessment, covariates were configured into three categories based on existing literature, known associations with the outcome of interest, and data availability within the database.: Model 1 adjusted for age and sex; Model 2 added adjustment for marital status, alcohol consumption, smoking status, and allergy history to Model 1; Model 3 further adjusted for waist circumference in addition to Model 2. R software (version 4.2.1; R Foundation for Statistical Computing),^1^ the R survey package (version 4.1–1), and Free Statistics software (version 2.1.1; Beijing Free Clinical Medical Technology Co., Ltd.) were used for analyses. In all analyses, a two-sided *p* < 0.05 was taken to indicate statistical significance.

### Subgroup analysis

To enhance the robustness of the study, subgroup analyses were conducted by age and gender to explore potential gender- and age-related differences.

## Results

### Characteristics of the study population

Among the 5,778 participants who completed the baseline survey, 5,667 met the inclusion criteria (mean age: 69.7 ± 8.6 years; mean BMI: 24.1 ± 3.3 kg/m^2^; 61.3% female).

Supplementary Table S1 presents the baseline prevalence of single and multiple metabolic abnormalities (“one high” to “five highs”) in the study population, stratified by sex and age groups: Among the baseline population, 98.2% of participants were married, 6% were smokers, and 2.8% had a history of alcohol consumption in the past year. The prevalence rates for hypertension, dyslipidemia, diabetes, hyperuricemia, hyperhomocysteinemia, and the presence of one, two, three, four, and five of these conditions were 62.5, 60.8, 36.4, 35.4, 83.5, 12.8, 24.6, 33.6, 21.6, 5.8%, respectively. According to Chinese standards (BMI ≥ 24 kg/m^2^), the prevalence of overweight was 37.4%, and that of obesity was 11.8%; according to WHO universal standards (BMI ≥ 25 kg/m^2^), the prevalence of overweight was 31.8%, and that of obesity was 4.7%. According to the WHO Asia-Pacific standard (BMI ≥ 23 kg/m^2^), the prevalence of overweight was 25.3%, and that of obesity was 36.5%.

The classification of individuals with five high-risk factors based on one of the three BMI standard thresholds is shown in Table 1. Hypertension (62.5%), dyslipidemia (60.8%), and hyperhomocysteinemia (83.5%) are the three metabolic problems with the highest incidence rate. Except for dyslipidemia, which had a *p* value of 0.126 (not significant) in the WHO universal standard, all other metabolic risks significantly increased with increasing BMI under the three BMI criteria (*p* < 0.05). About 61% of participants have ≥2 metabolic risks, and 33.6% have three risks simultaneously. As BMI increases, the proportion of individuals with three or more metabolic risks significantly increases (*p* < 0.001 for all BMI criteria). For example, in Chinese standards, 34.2% of people with a BMI ≥ 28 have three risks, while only 31.6% of people with a BMI < 24 have three risks. The Chinese standard (BMI ≥ 28 for obesity) and the WHO Asia Pacific standard (BMI ≥ 25 for obesity) are more sensitive in distinguishing metabolic risks.

**Table 1:** Metabolic risks characteristics according to the of overweight and obesity BMI threshold.

Metabolic risks	Cases	Incidence rate	Chinese BMI criteria				WHO BMI general criteria				WHO Asia Pacific BMI criteria			
			<24	24–28	≥28	*P*	<25	25–30	≥30	*P*	<23	23–25	≥25	*P*
	n	%	2,896	2,127	713		3,618	1810	308		2,179	1,439	2,118	
Hyperten-sion	3,541	62.5	1,622 (56.3)	1,428 (67.4)	491 (73.7)	<0.001	2086 (57.9)	1,256 (69.8)	199 (74.8)	<0.001	1,173 (54.2)	913 (63.6)	1,455 (70.4)	<0.001
Dyslipid-emia	3,448	60.8	1708 (59.3)	1,320 (62.3)	420 (63.1)	0.047	2,158 (59.9)	1,130 (62.8)	160 (60.2)	0.126	1,260 (58.2)	898 (62.5)	1,290 (62.4)	0.006
Diabetes	2064	36.4	983 (34.1)	809 (38.2)	272 (40.8)	<0.001	1,243 (34.5)	711 (39.5)	110 (41.4)	<0.001	733 (33.9)	510 (35.5)	821 (39.7)	<0.001
Hyperur-icemia	2005	35.4	815 (28.3)	877 (41.4)	313 (47)	<0.001	1,085 (30.1)	798 (44.3)	122 (45.9)	<0.001	570 (26.3)	515 (35.9)	920 (44.5)	<0.001
Hyperhomo-cysteinemia	4,730	83.5	2,305 (80)	1835 (86.6)	590 (88.6)	<0.001	2,909 (80.8)	1,591 (88.4)	230 (86.5)	<0.001	1727 (79.8)	1,182 (82.3)	1821 (88.1)	<0.001
Multiple metabolic risk						<0.001				<0.001				<0.001
0	84	1.5	70 (2.4)	11 (0.5)	3 (0.5)		75 (2.1)	8 (0.4)	1 (0.4)		59 (2.7)	16 (1.1)	9 (0.4)	
1	727	12.8	452 (15.7)	219 (10.3)	56 (8.4)		540 (15)	161 (8.9)	26 (9.8)		347 (16)	193 (13.4)	187 (9.1)	
2	1,395	24.6	829 (28.8)	450 (21.2)	116 (17.4)		992 (27.5)	354 (19.7)	49 (18.4)		664 (30.7)	328 (22.8)	403 (19.5)	
3	1904	33.6	911 (31.6)	765 (36.1)	228 (34.2)		1,183 (32.9)	636 (35.3)	85 (32)		669 (30.9)	514 (35.8)	721 (34.9)	
4	1,226	21.6	505 (17.5)	520 (24.5)	201 (30.2)		647 (18)	496 (27.6)	83 (31.2)		349 (16.1)	298 (20.8)	579 (28)	
5	331	5.8	114 (4)	155 (7.3)	62 (9.3)		164 (4.6)	145 (8.1)	22 (8.3)		77 (3.6)	87 (6.1)	167 (8.1)	

Continuous data are reported as the mean (standard deviation), and categorical data are reported as the number and percentage of participants. BMI, body mass index.

### Nonlinear relationship between BMI and the “five highs”

**Figure 2:**
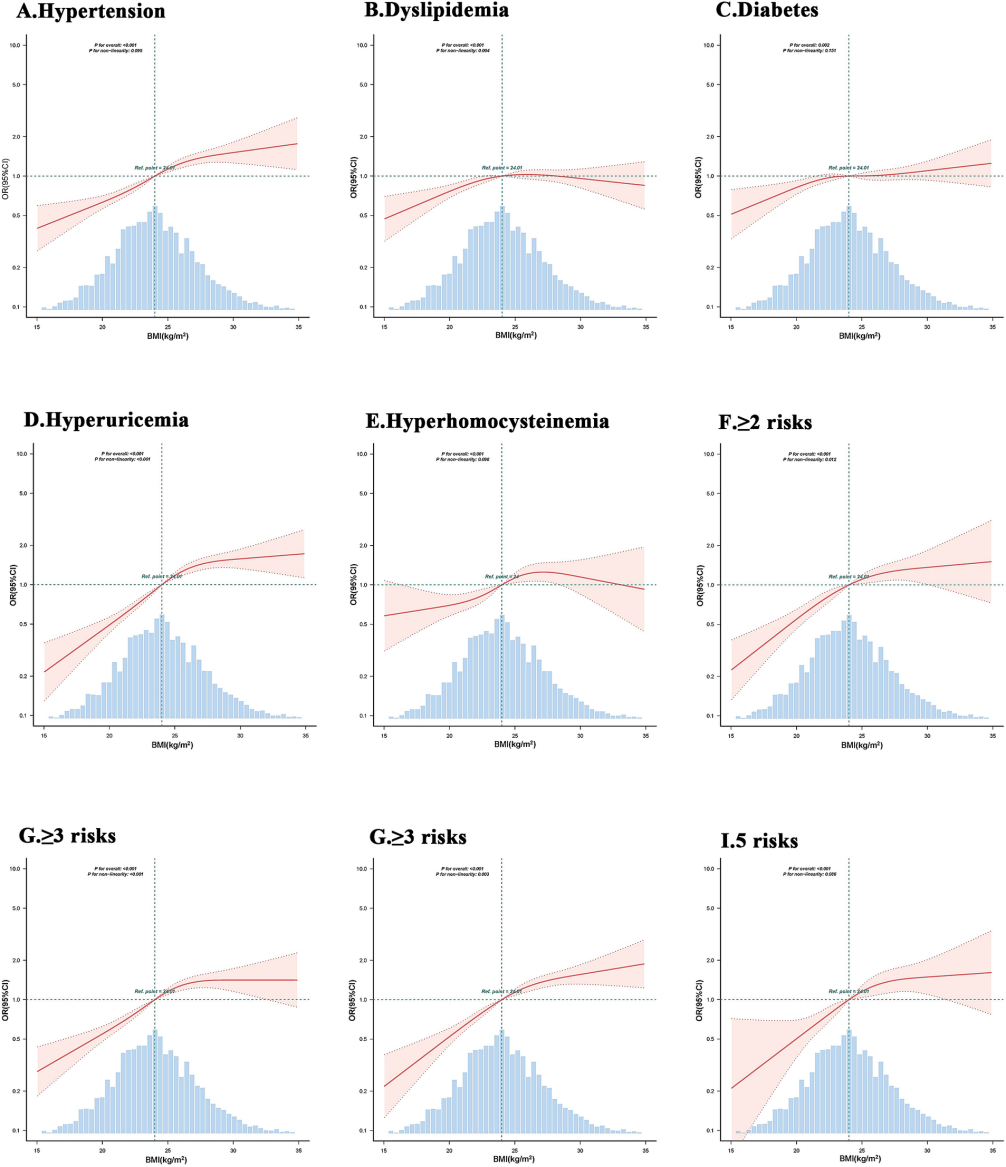
Logistic regression model with restricted cubic plots to evaluate the relationship between BMI and metabolic risk, and clustering. The model has been adjusted for age, gender, smoking, alcohol consumption, allergies, and waist circumference.

The results of the restricted cubic spline (RCS) analysis after multivariate adjustment indicate that BMI is significantly associated with hypertension, dyslipidemia, diabetes, hyperuricemia and hyperhomocysteinemia, as well as two-high, three-high, four-high, and five-high conditions. Among these relationships, BMI shows: A positive correlation with hypertension, diabetes, and five-high conditions; An inverted U-shaped relationship with dyslipidemia (initially increasing then decreasing, forming an inverted “U” pattern); a J-shaped relationship with hyperuricemia, two-high, three-high, and four-high conditions (showing little change or slight initial decrease, followed by a sharp increase);a linear correlation with hyperhomocysteinemia (Figure 2).

### Overweight/obesity status and risk of metabolic risk factors

Multivariate-adjusted logistic analysis showed that according to Chinese criteria, WHO general criteria, and Asia-Pacific criteria, overweight was associated with increased risks of hypertension, hyperuricemia, and multiple metabolic risks. Across all three criteria, overweight was robustly associated with elevated hypertension risk, and this association remained stable after adjusting for additional covariates. Both Chinese criteria [1.13 (1.01–1.28)] and WHO Asia-Pacific criteria [1.22 (1.06–1.4)] were associated with dyslipidemia, whereas no such association was found under WHO general criteria. WHO general criteria were associated with diabetes risk [1.15 (1.02–1.29)], while Chinese criteria and WHO Asia-Pacific criteria showed no such association. Overweight was consistently linked to hyperuricemia across all criteria and models. Chinese criteria [1.57 (1.27–1.94)] and WHO general criteria [1.92 (1.52–2.41)] were associated with hyperhomocysteinemia, whereas WHO Asia-Pacific criteria showed no such association (Table 2).

**Table 2:** Multiple logistic regression model revealed the risk of metabolic risk compared to normal and overweight.

Metabolic risks	Total	Incidence rate(%)	Model 1	*P*	Model 2	*P*	Model 3	*P*
Hypertension								
Chinese criteria	2120	1428 (67.4)	1.63 (1.45~1.83)	**<0.001**	1.63 (1.45~1.84)	**<0.001**	1.53 (1.36~1.73)	**<0.001**
WHO general criteria	1800	1256 (69.8)	1.7 (1.5~1.92)	**<0.001**	1.7 (1.51~1.92)	**<0.001**	1.6 (1.41~1.81)	**<0.001**
WHO Asia Pacific criteria	1436	913 (63.6)	1.5 (1.31~1.72)	**<0.001**	1.5 (1.31~1.73)	**<0.001**	1.49 (1.3~1.72)	**<0.001**
Dyslipidemia								
Chinese criteria	2120	1320 (62.3)	1.13 (1.01~1.27)	**0.035**	1.14 (1.01~1.28)	**0.031**	1.13 (1.01~1.28)	**0.037**
WHO general criteria	1800	1130 (62.8)	1.12 (0.99~1.26)	0.062	1.12 (0.99~1.26)	0.064	1.12 (0.99~1.26)	0.063
WHO Asia Pacific criteria	1436	898 (62.5)	1.22 (1.06~1.39)	**0.005**	1.22 (1.06~1.4)	**0.005**	1.22 (1.06~1.4)	**0.006**
Diabetes								
Chinese criteria	2120	809 (38.2)	1.17 (1.04~1.31)	**0.011**	1.17 (1.04~1.32)	**0.008**	1.09 (0.97~1.23)	0.138
WHO general criteria	1800	711 (39.5)	1.21 (1.08~1.36)	**0.001**	1.22 (1.08~1.37)	**0.001**	1.15 (1.02~1.29)	**0.025**
WHO Asia Pacific criteria	1436	510 (35.5)	1.06 (0.92~1.22)	0.433	1.05 (0.91~1.21)	0.483	1.02 (0.88~1.17)	0.822
Hyperuricemia								
Chinese criteria	2018	877 (43.5)	1.85 (1.64~2.09)	**<0.001**	1.85 (1.64~2.09)	**<0.001**	1.88 (1.65~2.13)	**<0.001**
WHO general criteria	1721	798 (46.4)	1.95 (1.73~2.2)	**<0.001**	1.95 (1.73~2.2)	**<0.001**	1.93 (1.71~2.19)	**<0.001**
WHO Asia Pacific criteria	1366	515 (37.7)	1.54 (1.33~1.79)	**<0.001**	1.54 (1.33~1.79)	**<0.001**	1.59 (1.36~1.85)	**<0.001**
Hyperhomocysteinemia								
Chinese criteria	1993	1835 (92.1)	1.59 (1.29~1.96)	**<0.001**	1.59 (1.29~1.96)	**<0.001**	1.57 (1.27~1.94)	**<0.001**
WHO general criteria	1704	1591 (93.4)	1.95 (1.55~2.44)	**<0.001**	1.95 (1.55~2.44)	**<0.001**	1.92 (1.52~2.41)	**<0.001**
WHO Asia Pacific criteria	1317	1182 (89.7)	1.15 (0.91~1.45)	0.234	1.15 (0.91~1.45)	0.25	1.13 (0.9~1.43)	0.301
Multiple metabolic risk								
≥2								
Chinese criteria	2120	1890 (89.2)	1.93 (1.63~2.28)	**<0.001**	1.91 (1.62~2.27)	**<0.001**	1.48 (1.21~1.8)	**<0.001**
WHO general criteria	1800	1631 (90.6)	2.11 (1.76~2.54)	**<0.001**	2.08 (1.73~2.5)	**<0.001**	1.51 (1.23~1.87)	**<0.001**
WHO Asia Pacific criteria	1436	1227 (85.4)	1.41 (1.17~1.7)	**<0.001**	1.41 (1.17~1.7)	**<0.001**	1.27 (1.03~1.57)	**0.027**
≥3								
Chinese criteria	2120	1440 (67.9)	1.9 (1.69~2.14)	**<0.001**	1.9 (1.69~2.14)	**<0.001**	1.5 (1.31~1.72)	**<0.001**
WHO general criteria	1800	1277 (70.9)	2.01 (1.78~2.27)	**<0.001**	2 (1.77~2.26)	**<0.001**	1.49 (1.3~1.72)	**<0.001**
WHO Asia Pacific criteria	1436	899 (62.6)	1.64 (1.43~1.88)	**<0.001**	1.64 (1.43~1.88)	**<0.001**	1.45 (1.25~1.69)	**<0.001**
≥4								
Chinese criteria	2120	675 (31.8)	1.7 (1.5~1.93)	**<0.001**	1.7 (1.5~1.94)	**<0.001**	1.61 (1.42~1.84)	**<0.001**
WHO general criteria	1800	641 (35.6)	1.92 (1.69~2.17)	**<0.001**	1.92 (1.69~2.17)	**<0.001**	1.82 (1.61~2.07)	**<0.001**
WHO Asia Pacific criteria	1436	385 (26.8)	1.47 (1.25~1.72)	**<0.001**	1.46 (1.25~1.71)	**<0.001**	1.4 (1.2~1.65)	**<0.001**
5								
Chinese criteria	2120	155 (7.3)	1.9 (1.48~2.44)	**<0.001**	1.92 (1.49~2.47)	**<0.001**	1.79 (1.39~2.3)	**<0.001**
WHO general criteria	1800	145 (8.1)	1.84 (1.46~2.32)	**<0.001**	1.84 (1.46~2.32)	**<0.001**	1.75 (1.39~2.21)	**<0.001**
WHO Asia Pacific criteria	1436	87 (6.1)	1.71 (1.25~2.35)	**0.001**	1.71 (1.25~2.35)	**0.001**	1.63 (1.19~2.24)	**0.003**

Model I: Adjusting for gender and age; Model II: Adjusting for gender, age, marriage status, smoking status, alcohol consumption, and allergy history; Model III: Adjust for gender, age, marriage status, smoking status, alcohol consumption, allergy history, and waist circumference. Bold font indicates *P* < 0.05.

Table 3 summarizes a multiple logistic regression model that revealed that the risk of metabolic risk compared to normal and obesity. According to Chinese criteria, WHO general criteria, and Asia-Pacific criteria, obesity was associated with increased risks of hypertension, hyperuricemia, four-high, and five-high conditions. WHO Asia-Pacific criteria were associated with increased dyslipidemia risk [1.19 (1.05–1.35)], while Chinese criteria and WHO general criteria showed no such association. Chinese criteria and WHO Asia-Pacific criteria were associated with increased risks of diabetes, hyperhomocysteinemia, two-high and three-high conditions, whereas WHO general criteria showed no such association.

**Table 3:** Multiple logistic regression model revealed the risk of metabolic risk compared to normal and obesity.

Metabolic risks	Total	Incidence rate(%)	Model 1	*P*	Model 2	*P*	Model 3	*P*
Hypertension								
Chinese criteria	666	491 (73.7)	2.23 (1.85~2.7)	**<0.001**	2.25 (1.86~2.72)	**<0.001**	2.11 (1.74~2.55)	**<0.001**
WHO general criteria	266	199 (74.8)	2.21 (1.66~2.94)	**<0.001**	2.22 (1.67~2.96)	**<0.001**	2.11 (1.58~2.82)	**<0.001**
WHO Asia Pacific criteria	2066	1455 (70.4)	2.06 (1.81~2.34)	**<0.001**	2.07 (1.82~2.35)	**<0.001**	1.94 (1.7~2.21)	**<0.001**
Dyslipidemia								
Chinese criteria	666	420 (63.1)	1.14 (0.96~1.36)	0.13	1.15 (0.96~1.37)	0.129	1.14 (0.95~1.36)	0.159
WHO general criteria	266	160 (60.2)	0.98 (0.76~1.26)	0.855	0.98 (0.76~1.26)	0.87	0.97 (0.75~1.25)	0.808
WHO Asia Pacific criteria	2066	1290 (62.4)	1.19 (1.05~1.34)	**0.007**	1.19 (1.05~1.35)	**0.006**	1.19 (1.05~1.35)	**0.007**
Diabetes								
Chinese criteria	666	272 (40.8)	1.27 (1.07~1.51)	**0.007**	1.28 (1.07~1.53)	**0.006**	1.2 (1.01~1.43)	**0.044**
WHO general criteria	266	110 (41.4)	1.27 (0.99~1.64)	**0.064**	1.28 (0.99~1.65)	0.063	1.2 (0.93~1.55)	0.169
WHO Asia Pacific criteria	2066	821 (39.7)	1.25 (1.1~1.42)	**0.001**	1.25 (1.1~1.42)	**0.001**	1.16 (1.02~1.32)	**0.023**
Hyperuricemia								
Chinese criteria	647	313 (48.4)	2.42 (2.02~2.9)	**<0.001**	2.42 (2.02~2.9)	**<0.001**	2.42 (2.01~2.9)	**<0.001**
WHO general criteria	258	122 (47.3)	2.14 (1.65~2.78)	**<0.001**	2.14 (1.65~2.78)	**<0.001**	2.09 (1.61~2.73)	**<0.001**
WHO Asia Pacific criteria	1979	920 (46.5)	2.37 (2.07~2.71)	**<0.001**	2.37 (2.07~2.71)	**<0.001**	2.38 (2.07~2.73)	**<0.001**
Hyperhomocysteinemia								
Chinese criteria	637	590 (92.6)	2.02 (1.45~2.81)	**<0.001**	2.01 (1.44~2.8)	**<0.001**	1.96 (1.4~2.76)	**<0.001**
WHO general criteria	253	230 (90.9)	1.62 (1.02~2.55)	**0.039**	1.61 (1.02~2.54)	**0.042**	1.56 (0.98~2.48)	0.061
WHO Asia Pacific criteria	1957	1821 (93.1)	2 (1.59~2.52)	**<0.001**	2 (1.58~2.52)	**<0.001**	1.96 (1.55~2.48)	**<0.001**
Multiple metabolic risk								
≥2								
Chinese criteria	666	607 (91.1)	2.6 (1.95~3.47)	**<0.001**	2.54 (1.9~3.39)	**<0.001**	1.74 (1.25~2.44)	**0.001**
WHO general criteria	266	239 (89.8)	2.1 (1.39~3.17)	**<0.001**	2.06 (1.37~3.12)	**0.001**	1.25 (0.79~1.97)	0.333
WHO Asia Pacific criteria	2066	1870 (90.5)	2.41 (2~2.9)	**<0.001**	2.37 (1.97~2.86)	**<0.001**	1.69 (1.34~2.14)	**<0.001**
≥3								
Chinese criteria	666	491 (73.7)	2.61 (2.16~3.15)	**<0.001**	2.58 (2.14~3.12)	**<0.001**	1.75 (1.4~2.19)	**<0.001**
WHO general criteria	266	190 (71.4)	2.13 (1.62~2.8)	**<0.001**	2.12 (1.61~2.79)	**<0.001**	1.26 (0.93~1.72)	0.138
WHO Asia Pacific criteria	2066	1467 (71)	2.47 (2.17~2.8)	**<0.001**	2.45 (2.16~2.79)	**<0.001**	1.82 (1.55~2.15)	**<0.001**
≥4								
Chinese criteria	666	263 (39.5)	2.44 (2.03~2.92)	**<0.001**	2.43 (2.03~2.91)	**<0.001**	2.27 (1.89~2.73)	**<0.001**
WHO general criteria	266	105 (39.5)	2.31 (1.78~2.99)	**<0.001**	2.31 (1.78~3)	**<0.001**	2.18 (1.68~2.83)	**<0.001**
WHO Asia Pacific criteria	2066	746 (36.1)	2.31 (2.01~2.66)	**<0.001**	2.31 (2.01~2.66)	**<0.001**	2.16 (1.88~2.49)	**<0.001**
≥5								
Chinese criteria	666	62 (9.3)	2.52 (1.82~3.49)	**<0.001**	2.53 (1.83~3.51)	**<0.001**	2.4 (1.73~3.33)	**<0.001**
WHO general criteria	266	22 (8.3)	1.92 (1.2~3.06)	**0.006**	1.92 (1.2~3.07)	**0.006**	1.84 (1.15~2.95)	**0.011**
WHO Asia Pacific criteria	2066	167 (8.1)	2.37 (1.8~3.14)	**<0.001**	2.37 (1.8~3.14)	**<0.001**	2.22 (1.67~2.94)	**<0.001**

Model I: Adjusting for gender and age; Model II: Adjusting for gender, age, marriage status, smoking status, alcohol consumption, and allergy history; Model III: Adjust for gender, age, marriage status, smoking status, alcohol consumption, allergy history, and waist circumference. Bold font indicates *P* < 0.05.

### Subgroup analysis by age and gender

**Figure 3:**
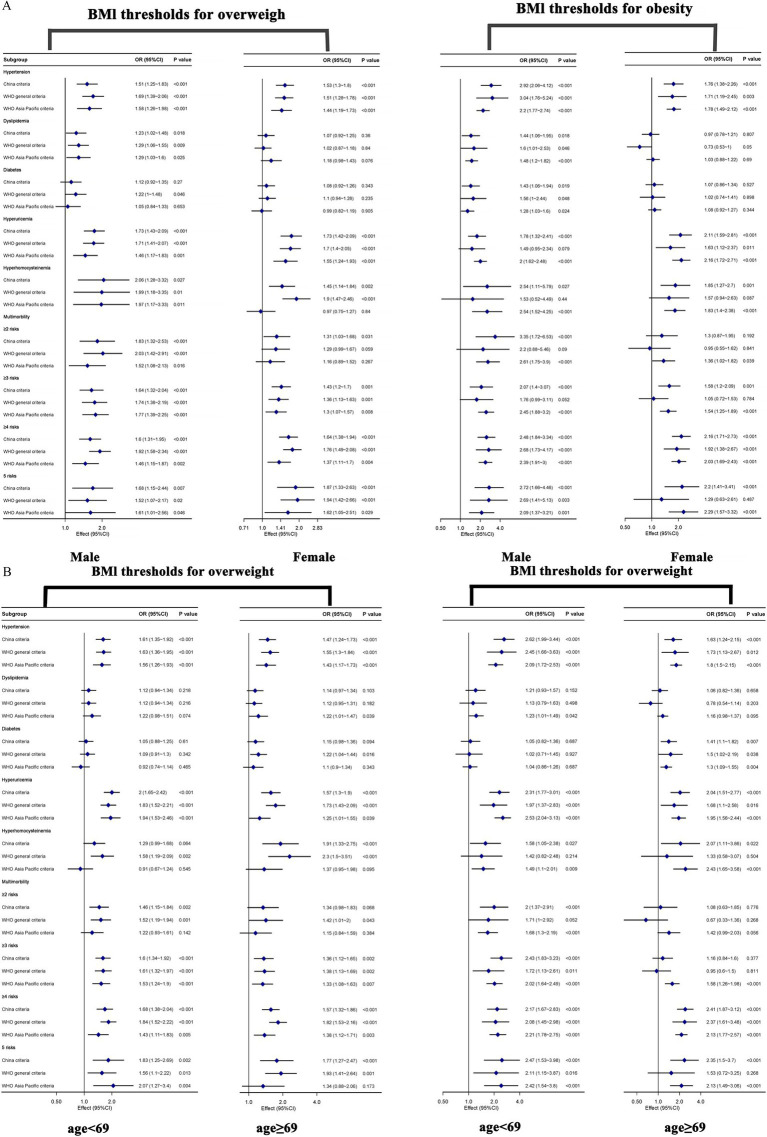
**(A)** Subgroup analysis of three BMI threshold criteria revealed the association between overweight/obesity and metabolic factors (grouped by gender). Adjust for gender, age, marriage status, smoking status, alcohol consumption, allergy history, and waist circumference. **(B)** Subgroup analysis of three BMI threshold criteria revealed the association between overweight/obesity and metabolic factors (grouped by age). Adjust for gender, age, marriage status, smoking status, alcohol consumption, allergy history, and waist circumference.

Supplementary Table S2 shows subgroup analyses by sex, revealing sex-specific associations between BMI and metabolic abnormalities: Obesity conferred a higher risk for hypertension in males (e.g., Chinese criteria: OR = 2.92 vs. 1.76), with stronger trends and borderline significant gender interaction. The association between BMI and dyslipidemia existed almost exclusively in males, where both overweight and obesity significantly increased dyslipidemia risk with strong dose–response trends, with WHO general criteria obesity showing an OR of [1.6 (1.01–2.53)]. In contrast, virtually all associations were non-significant in females. Diabetes risk was primarily associated with BMI in males, with no significant associations observed in females (P for interaction >0.05), indicating significant gender interaction. For hyperuricemia, although associations were significant in both sexes, the risk from obesity was higher in females (OR 2.11–2.16 vs. 1.78–2.00). Notably, baseline prevalence in males (21.2%) was substantially lower than in females (42.9%), yet elevated BMI conferred greater absolute risk increases in females. Regarding hyperhomocysteinemia, risks from overweight and obesity were more consistent in males, whereas in females only obesity emerged as a significant risk factor, with Chinese criteria showing an OR of [1.85 (1.27–2.7)]. For the risk of developing two or more metabolic factors, males showed greater sensitivity to BMI elevation, with both overweight and obesity significantly increasing risk. Female associations were weaker, particularly for obesity status, where risks were non-significant under certain criteria (P for interaction <0.05). When three or more metabolic factors clustered together, gender interaction was only significant under WHO Asia-Pacific criteria. In both males and females, overweight and obesity generally increased risk significantly, though female obesity showed non-significant associations under WHO general criteria (*p* = 0.784). For the clustering of four or more and all five metabolic factors, gender interactions were non-significant. Except for non-significant obesity risk in females under WHO general criteria, overweight and obesity significantly and substantially increased risks in the vast majority of cases, with equally detrimental effects in both sexes (Figure 3A).

Subgroup analysis by age group showed that under the Chinese criteria, the risk of hypertension associated with obesity was significantly higher in the young group [2.62 (1.99–3.44)] (P for interaction = 0.035). All criteria showed a strong correlation between BMI and hypertension, with a larger increase in risk in the young group. The WHO Asia-Pacific criteria were the only criteria that could stably reveal a significant association between BMI and dyslipidemia, especially in the young group (P for interaction >0.05). Contrary to the pattern of hypertension, the strong association between overweight/obesity and diabetes was mainly concentrated in the older group (≥69 years), and although the age interaction was not significant, the pattern was highly consistent. BMI had a consistently strong positive correlation with hyperuricemia across all age groups (P for interaction = 0.035), with the highest risk in the young group under the WHO Asia-Pacific criteria [2.53 (2.04–3.13)]. The strong association between overweight/obesity and elevated homocysteine was mainly concentrated in the older group (≥69 years) (P for interaction = 0.052). Overweight/obesity was a strong risk factor for having two or three metabolic risk factors in the young group, while this association was greatly weakened or disappeared in the older group. In the case of clustering of four or more and five metabolic risk factors, obesity became an equal threat across all age groups, with a large and consistent risk and no age interaction (Figure 3B and Supplementary Table S3).

## Discussion

This study systematically compared the efficacy of three body mass index (BMI) thresholds—the Chinese criteria, the WHO universal standard, and the WHO Asia-Pacific standard—in identifying the “five highs” (hypertension, dyslipidemia, diabetes, hyperuricemia, and hyperhomocysteinemia) and their clustered risk, based on cross-sectional data from basic public health examinations in Western Guangdong, China. Our core findings indicate that among the population of Western Guangdong—characterized by unique geographical and lifestyle traits—the Chinese BMI standards (overweight ≥24 kg/m^2^, obese ≥28 kg/m^2^) demonstrate greater sensitivity in identifying individuals at risk for metabolic abnormalities compared to both the WHO General Standard and the WHO Asia-Pacific Standard. Furthermore, these Chinese standards exhibit a stronger association with the clustered state of the “Five Highs” (7, 30, 31). This conclusion not only provides direct guidance for public health strategies in Western Guangdong but also offers crucial evidence-based support for developing regionally tailored chronic disease prevention and control guidelines in China.

The primary finding of this study is that the Chinese BMI standard demonstrates exceptional risk identification capability among populations in Western Guangdong. Extensive prior research confirms that compared to Caucasians, Asian populations exhibit higher body fat content—particularly visceral fat—at equivalent BMI levels. This results in significantly elevated metabolic disease risks at lower BMI thresholds (32–35). The WHO Asia-Pacific standard (overweight ≥23 kg/m^2^) was proposed precisely to address this biological difference, aiming to enhance risk detection sensitivity for Asian populations (36). However, our findings further suggest that the Chinese standard, developed by integrating large-scale epidemiological data from China, may more accurately align with the pathophysiological characteristics of Chinese populations, including those in Western Guangdong.

In our analysis, when applying the WHO universal standard (overweight ≥25 kg/m^2^), a significant number of individuals who have already exhibited clinical metabolic abnormalities may be erroneously classified as “normal weight,” thereby missing the optimal window for early intervention (37, 38). For instance, individuals with a BMI between 24 and 24.9 kg/m^2^ in this study would be classified as “overweight” under Chinese standards (39, 40). Their prevalence of hypertension, dyslipidemia, and other conditions is already significantly higher than that of individuals with a BMI < 24 kg/m^2^ (41, 42). If this group were deemed healthy under the WHO universal standard, it would create a “blind spot” in the coverage of public health prevention resources. Based on the nonlinear relationships revealed by restricted cubic spline analysis, this study further explored their potential biological mechanisms: the positive correlations between BMI and hypertension, diabetes, and the “five-high” clustering may stem from the cumulative effects of obesity-related insulin resistance and chronic inflammatory states; the inverted U-shaped relationship with dyslipidemia might reflect a dynamic balance between early-stage deterioration of lipid metabolism and compensatory dysfunction in hepatic synthesis during severe obesity; the J-shaped relationship with hyperuricemia and clustering of multiple metabolic abnormalities suggests the presence of a critical threshold for metabolic compensatory mechanisms, whereby exceeding a certain BMI level triggers cascading reactions of multi-system metabolic disorders, such as renal uric acid excretion failure; as for the linear association with hyperhomocysteinemia, it may be closely related to insufficient intake of folate and B vitamins among obese populations, combined with impaired activity of relevant metabolic enzymes.

Logistic regression analysis in this study revealed that, relative to the normal weight group, the adjusted odds ratios (OR values) for the overweight and obese groups classified according to Chinese standards showed the most pronounced gradient increase and highest numerical values in association with the risk of multiple disease clusters including “two highs” and above. This indicates that Chinese standards not only identify more high-risk individuals but also establish the strongest link between the defined states of ‘overweight’ and “obesity” and metabolic risk. Restricted Cubic Spline (RCS) analysis corroborates this finding. The dose–response curve for BMI and the risk of clustering five metabolic disorders exhibits the most pronounced slope change near the 24 and 28 kg/m^2^ thresholds set by the Chinese standards, suggesting these two cut-off points serve as effective critical values for risk stratification. This aligns with nationwide findings linking BMI to cardiovascular risk factor clustering and has been validated in Western Guangdong (43).

Subgroup analysis shows that gender differences may be mediated by a variety of factors: Men are more sensitive to increased BMI: Men are more prone to visceral fat accumulation (abdominal obesity), which has high lipolytic activity and is more likely to lead to lipotoxicity, insulin resistance, and systemic inflammation. Androgen levels in men may also play a role by affecting fat distribution and metabolic pathways. Hyperuricemia risk in women: Estrogen in premenopausal women promotes the excretion of uric acid. Therefore, at the same BMI level, the risk of hyperuricemia in women (especially premenopausal) is usually lower than that in men. The specific risk observed in women in this study needs to be further explored in combination with dietary structure (such as intake of sugary drinks and high-purine foods) and possible changes in hormone levels. In addition, women’s fat distribution is more inclined to subcutaneous fat, which has relatively low metabolic activity, and its impact on uric acid production may be different from that in men.

This study extends beyond isolated metabolic disorders by innovatively incorporating hyperhomocysteinemia into its analytical framework, establishing a more comprehensive metabolic risk assessment model termed the “Five Highs.” Hyperhomocysteinemia has gained widespread recognition in recent years as an independent cardiovascular risk factor, closely associated with obesity, insulin resistance, hypertension, and other conditions (44–46). In recent years, a number of studies have shown that even if the level of homocysteine (Hcy) is within the range of 10–15 μmol/L, the risk of cardiovascular disease and neurodegenerative disease has increased significantly. Using ≥10 μmol/L significantly increases the detection rate of HHcy, resulting in more subjects being classified as “abnormal.” While this may enhance statistical significance, it could also include some individuals who are actually at lower risk, leading to overdiagnosis and overtreatment. The concept of the “Five Highs” underscores the systemic nature and synergistic hazards of metabolic abnormalities (47). Obesity, as a central component, drives the onset and progression of hypertension, dyslipidemia, hyperglycemia, and hyperuricemia by inducing and exacerbating insulin resistance. These factors then interact to form a vicious cycle, ultimately leading to severe endpoints such as atherosclerotic cardiovascular disease (ASCVD), renal impairment, and cognitive dysfunction (48).

Our findings indicate that as BMI levels increase—particularly when measured by Chinese standards—the prevalence of “two highs,” “three highs,” and even “five highs” rises exponentially. This means that with each unit increase in BMI, individuals face not merely an elevated risk of a single disease, but a compound health threat stemming from the synergistic effects of multiple risk factors. Therefore, in clinical practice, overweight or obese individuals should undergo comprehensive metabolic risk screening—including lipid, uric acid, and homocysteine levels—rather than merely achieving target blood pressure and blood glucose levels. The lower BMI cutoffs in the Chinese standard classify more individuals as “overweight” or “obese,” thereby increasing the sensitivity for detecting at-risk individuals but potentially at the cost of lower specificity (more false positives). The WHO standards, with higher cutoffs, may be more specific but risk missing a segment of the population with metabolically unhealthy normal weight, particularly relevant for the Chinese population. In clinical practice, the choice of standard involves a trade-off. For screening purposes in a public health context, the higher sensitivity of the Chinese standard may be preferable to cast a wider net. For resource-intensive interventions, a more specific standard might be used to prioritize individuals. This study underscores that using the Chinese BMI standard as a trigger point for initiating comprehensive metabolic assessment offers greater cost-effectiveness and clinical predictive value.

For public health policymakers, this finding implies that obesity prevention and control should be placed at the core of primary prevention strategies for metabolic diseases (49, 50). In Western Guangdong, health education, community screening, and intervention measures should prioritize individuals with a BMI ≥ 24 kg/m^2^. Given the region’s unique subtropical maritime climate and dietary habits characterized by high salt and fat intake alongside substantial seafood consumption, the drivers of obesity and the “five highs” (high blood pressure, high blood sugar, high blood lipids, high blood pressure, and high blood sugar) may exhibit both universal and region-specific characteristics (51, 52). Future intervention strategies should be tailored precisely to local conditions within the framework of national guidelines.

First, this study focuses on a specific population in Western Guangdong, China, filling a gap in research on the association between BMI standards and the spectrum of metabolic diseases in this region. The unique genetic background and lifestyle of this area lend the study’s conclusions significant regional representativeness and relevance. Second, this study developed the comprehensive outcome indicator “five highs,” which are highly prevalent in Chinese populations, extending beyond the traditional “three highs” or metabolic syndrome framework. This approach provides a more holistic portrayal of obesity-related multisystem metabolic disorders, deepening our understanding of obesity’s health risks. Third, the study employed rigorous head-to-head comparisons, directly evaluating three internationally recognized BMI standards within the same research framework. This provides direct, compelling evidence regarding the relative merits of different standards within specific populations. Finally, the study features a large sample size, standardized data collection, and scientifically sound statistical analysis methods, ensuring the reliability and validity of the findings.

Although this study yielded important findings, several limitations exist. First, as a cross-sectional design, it cannot establish a causal relationship between BMI and the “five highs” but only indicates the strength of association. Second, the study population is limited to the western part of Guangdong, and the average age is older, so we need to be cautious when extrapolating the results to other parts of China (such as the northern inland areas) and young and middle-aged people. Differences in dietary patterns, climatic conditions, and genetic background in different regions and ages may contribute to the heterogeneity of optimal BMI thresholds. Thirdly, although we have adjusted for major confounding factors such as age and gender, residual confounding factors (including physical activity levels, specific dietary patterns, and socioeconomic status) may not have been fully controlled, which could lead to bias and introduce residual confounding factors in metabolic risk assessment. Fourth, while the “five highs” diagnostic criteria employed in this study align with current guidelines, some indicators (e.g., the diagnostic cutoff for hyperhomocysteinemia) remain subject to academic debate. The adoption of different standards may influence the findings.

## Conclusion

In summary, this study provides strong evidence that the Chinese BMI standard (overweight ≥24 kg/m^2^, obesity ≥28 kg/m^2^) is the most suitable and effective tool for assessing the “five highs” and their clustered risk among the population in Western Guangdong, China. The application of this standard facilitates earlier and more accurate identification of high-risk individuals for metabolic diseases, holding critical value for optimizing regional public health resource allocation and formulating precise primary prevention strategies. Future prospective follow-up studies are needed to clarify the predictive capabilities of different BMI standards for the “five highs” and cardiovascular endpoints.

Drawing on the findings of this study, we propose that public health departments in southern China integrate the Chinese BMI criteria into community screening and risk assessment protocols. This move is aimed at bolstering the early detection of individuals at risk for the “five highs.” Moving forward, research efforts could prioritize the development of low-cost, high-compliance screening models. Empirical validation through pilot programs would be instrumental in refining these approaches, ultimately enhancing the practical applicability and real-world effectiveness of the BMI criteria over time. Developing gender- and age-specific BMI action plans, and using electronic health records to automate risk stratification and follow-up. Additionally, in health education initiatives, we advocate leveraging the more stringent cut-off points of the Chinese BMI criteria to underscore the metabolic risks associated with even modest weight gain. To fortify the evidence base, we are set to embark on the following research endeavors: validating the predictive accuracy of the Chinese BMI criteria for metabolic outcomes and hard endpoints like cardiovascular events through prospective cohort studies; and delving into the genetic and environmental underpinnings of gender differences.

## Data Availability

The raw data supporting the conclusions of this article will be made available by the authors, without undue reservation.

## Footnotes

1
http://www.Rproject.org
