# Correction: Comparative analysis of three BMI cutoffs for five metabolic abnormalities in the population of Western Guangdong, China

**DOI:** 10.3389/fnut.2026.1835814

**Published:** 2026-04-07

**Authors:** Dengwen Pang, Meizhu Chen, Chengwen Xiao, Ruijian Zhuang, Chengyu Kong, Mengyuan Xiao

**Affiliations:** 1 Department of Cardiovascular Medicine Center, Affiliated Hospital of Guangdong Medical University, Zhanjiang, China; 2 Department of Assets and Equipment Management, Guangdong Medical University, Zhanjiang, Guangdong, China; 3 Dongxin Street Community Health Service Center, Zhanjiang, Guangdong, China; 4 The First Clinical College, Guangdong Medical University, Zhanjiang, Guangdong, China

**Keywords:** body mass index, metabolic risk, “5 highs”, obesity, Western Guangdong population

In the originally published article, the affiliation of the corresponding author Mengyuan Xiao was incorrectly listed as the fourth affiliation. Due to institutional requirements at the Hospital of Guangdong Medical University, Dr. Xiao's affiliation, the Department of Cardiovascular Medicine Center, Affiliated Hospital of Guangdong Medical University, Zhanjiang, China, must be listed as the first affiliation for funding reimbursement and administrative purposes. The correct affiliation list is as follows:

Dengwen Pang^2^, Meizhu Chen^3^, Chengwen Xiao^4^, Ruijian Zhuang^4^, Chengyu Kong^4^ and Mengyuan Xiao^1*^

1 Department of Cardiovascular Medicine Center, Affiliated Hospital of Guangdong Medical University, Zhanjiang, China

2 Department of Assets and Equipment Management, Guangdong Medical University, Zhanjiang, Guangdong, China

3 Dongxin Street Community Health Service Center, Zhanjiang, Guangdong, China

4 The First Clinical College, Guangdong Medical University, Zhanjiang, Guangdong, China

The original version of this article has been updated.

