# Supplementary material for: Comparative analysis of three BMI cutoffs for five metabolic abnormalities in the population of Western Guangdong, China
Source: Front Nutr. 2025 Dec 3;12:1732345. doi: 10.3389/fnut.2025.1732345 (PMC12708589; doi:10.3389/fnut.2025.1732345)
Supplement: Supplementary file 1 [file Table_1.docx]

**Table S1.Clinical characteristics of the study population.**

| **Variables** | **Total**  **(n = 5667)** | **Male**  **(n = 2193)** | **Female**  **(n = 3474)** | ***P*** |
| --- | --- | --- | --- | --- |
| **Age, (years)** | 69.7 ± 8.6 | 70.2 ± 8.6 | 69.5 ± 8.6 | 0.002 |
| **Marriage status, n (%)** | 4040 (98.2) | 1556 (97.9) | 2484 (98.3) | 0.384 |
| **Smoking status, n (%)** | 338 ( 6.0) | 330 (15) | 8 (0.2) | < 0.001 |
| **Alcohol consumption, n (%)** | 159 ( 2.8) | 152 (6.9) | 7 (0.2) | < 0.001 |
| **Allergy history,n(%)** | 165 ( 2.9) | 48 (2.2) | 117 (3.4) | 0.036 |
| **Weight Mean ± SD** | 61.4 ± 10.3 | 66.8 ± 9.8 | 58.0 ± 9.1 | < 0.001 |
| **High, Mean ± SD** | 159.4 ± 8.1 | 166.4 ± 5.9 | 155.0 ± 5.8 | < 0.001 |
| **BMI, Mean ± SD** | 24.1 ± 3.3 | 24.1 ± 3.1 | 24.1 ± 3.4 | 0.676 |
| **WC, Median (IQR)** | 86.0 (80.0, 91.0) | 88.0 (82.0, 94.0) | 84.0 (78.7, 89.0) | < 0.001 |
| **Hypertension, n (%)** | 3541 (62.5) | 1347 (61.4) | 2194 (63.2) | 0.19 |
| **Dyslipidemia, n (%)** | 3448 (60.8) | 1243 (56.7) | 2205 (63.5) | < 0.001 |
| **Diabetes, n (%)** | 2064 (36.4) | 785 (35.8) | 1279 (36.8) | 0.437 |
| **Hyperuricemia, n (%)** | 2005 (35.4) | 1046 (47.7) | 959 (27.6) | < 0.001 |
| **Hyperhomocysteinemia, n (%)** | 4730 (83.5) | 1931 (88.1) | 2799 (80.6) | < 0.001 |
| **Multiple metabolic risk , n (%)** |  |  |  | < 0.001 |
| **0** | 84 ( 1.5) | 18 (0.8) | 66 (1.9) |  |
| **1** | 727 (12.8) | 261 (11.9) | 466 (13.4) |  |
| **2** | 1395 (24.6) | 496 (22.6) | 899 (25.9) |  |
| **3** | 1904 (33.6) | 725 (33.1) | 1179 (33.9) |  |
| **4** | 1226 (21.6) | 541 (24.7) | 685 (19.7) |  |
| **5** | 331 ( 5.8) | 152 (6.9) | 179 (5.2) |  |
| **Chinese BMI Criteria, n (%)** |  |  |  | 0.018 |
| **<24 kg/m^2^** | 2881 (50.8) | 1104 (50.3) | 1777 (51.2) |  |
| **24-28 kg/m^2^** | 2120 (37.4) | 859 (39.2) | 1261 (36.3) |  |
| **≥28 kg/m^2^** | 666 (11.8) | 230 (10.5) | 436 (12.6) |  |
| **WHO BMI general Criteria, n (%)** |  |  |  | 0.138 |
| **<23 kg/m^2^** | 3601 (63.5) | 1412 (64.4) | 2189 (63) |  |
| **23-25 kg/m^2^** | 1800 (31.8) | 693 (31.6) | 1107 (31.9) |  |
| **≥25 kg/m^2^** | 266 ( 4.7) | 88 (4) | 178 (5.1) |  |
| **WHO Asia Pacific BMI Criteria, n (%)** |  |  |  | < 0.001 |
| **<25 kg/m^2^** | 2165 (38.2) | 795 (36.3) | 1370 (39.4) |  |
| **25-30 kg/m^2^** | 1436 (25.3) | 617 (28.1) | 819 (23.6) |  |
| **≥30 kg/m^2^** | 2066 (36.5) | 781 (35.6) | 1285 (37) |  |

Continuous data are reported as the mean (standard deviation), and categorical data are reported as the number and percentage of participants.BMI, body mass index.
