# Supplementary material for: Comparative analysis of three BMI cutoffs for five metabolic abnormalities in the population of Western Guangdong, China
Source: Front Nutr. 2025 Dec 3;12:1732345. doi: 10.3389/fnut.2025.1732345 (PMC12708589; doi:10.3389/fnut.2025.1732345)
Supplement: Supplementary file 2 [file Table_2.docx]

**Table S2(a) Subgroup Analysis by Gender: Association Between Different BMI Criteria and Hypertension.**

| **Hypetension** | **Male** | | | **Female** | | | **P for interaction** |
| --- | --- | --- | --- | --- | --- | --- | --- |
|  | **Incidence rate(%)** | **OR** | ***P*** | **Incidence rate(%)** | **OR** | ***P*** |  |
| **Chinese Criteria** |  |  |  |  |  |  | 0.032 |
| Chinese Criteria | 600 (54.3) | 1(Ref) |  | 1022 (57.5) | 1(Ref) |  |  |
| WHO general Criteria | 566 (65.9) | 1.51 (1.25~1.83) | <0.001 | 862 (68.4) | 1.53 (1.3~1.8) | <0.001 |  |
| WHO Asia Pacific Criteria | 181 (78.7) | 2.92 (2.06~4.12) | <0.001 | 310 (71.1) | 1.76 (1.38~2.26) | <0.001 |  |
| **WHO general Criteria** |  |  |  |  |  |  | 0.134 |
| Chinese Criteria | 794 (56.2) | 1(Ref) |  | 1292 (59) | 1(Ref) |  |  |
| WHO general Criteria | 482 (69.6) | 1.69 (1.39~2.06) | <0.001 | 774 (69.9) | 1.51 (1.28~1.78) | <0.001 |  |
| WHO Asia Pacific Criteria | 71 (80.7) | 3.04 (1.76~5.24) | <0.001 | 128 (71.9) | 1.71 (1.19~2.45) | 0.003 |  |
| **WHO Asia Pacific Criteria** |  |  |  |  |  |  | 0.215 |
| Chinese Criteria | 405 (50.9) | 1(Ref) |  | 768 (56.1) | 1(Ref) |  |  |
| WHO general Criteria | 389 (63) | 1.58 (1.26~1.98) | <0.001 | 524 (64) | 1.44 (1.19~1.73) | <0.001 |  |
| WHO Asia Pacific Criteria | 553 (70.8) | 2.2 (1.77~2.74) | <0.001 | 902 (70.2) | 1.78 (1.49~2.12) | <0.001 |  |

Adjust for gender, age, marriage status, smoking status, alcohol consumption, allergy history, and waist circumference.

**Table S2(b) Subgroup Analysis by Gender: Association Between Different BMI Criteria and Dyslipidemia.**

| **Dyslipidemia** | **Male** | | | **Female** | | | **P for interaction** |
| --- | --- | --- | --- | --- | --- | --- | --- |
|  | **Incidence rate(%)** | **OR** | ***P*** | **Incidence rate(%)** | **OR** | ***P*** |  |
| **Chinese Criteria** |  |  |  |  |  |  | 0.061 |
| Chinese Criteria | 595 (53.9) | 1(Ref) |  | 1113 (62.6) | 1(Ref) |  |  |
| WHO general Criteria | 502 (58.4) | 1.23 (1.02~1.48) | 0.032 | 818 (64.9) | 1.07 (0.92~1.25) | 0.36 |  |
| WHO Asia Pacific Criteria | 146 (63.5) | 1.44 (1.06~1.95) | 0.018 | 274 (62.8) | 0.97 (0.78~1.21) | 0.807 |  |
| **WHO general Criteria** |  |  |  |  |  |  | 0.004 |
| Chinese Criteria | 767 (54.3) | 1(Ref) |  | 1391 (63.5) | 1(Ref) |  |  |
| WHO general Criteria | 418 (60.3) | 1.29 (1.06~1.55) | 0.009 | 712 (64.3) | 1.02 (0.87~1.18) | 0.84 |  |
| WHO Asia Pacific Criteria | 58 (65.9) | 1.6 (1.01~2.53) | 0.046 | 102 (57.3) | 0.73 (0.53~1) | 0.05 |  |
| **WHO Asia Pacific Criteria** |  |  |  |  |  |  | 0.013 |
| Chinese Criteria | 411 (51.7) | 1(Ref) |  | 849 (62) | 1(Ref) |  |  |
| WHO general Criteria | 356 (57.7) | 1.29 (1.03~1.6) | 0.025 | 542 (66.2) | 1.18 (0.98~1.43) | 0.076 |  |
| WHO Asia Pacific Criteria | 476 (60.9) | 1.48 (1.2~1.82) | <0.001 | 814 (63.3) | 1.03 (0.88~1.22) | 0.69 |  |

Adjust for gender, age, marriage status, smoking status, alcohol consumption, allergy history, and waist circumference.

**Table S2(c) Subgroup Analysis by Gender: Association Between Different BMI Criteria and Diabetes.**

| **Diabetes** | **Male** | | | **Female** | | | **P for interaction** |
| --- | --- | --- | --- | --- | --- | --- | --- |
|  | **Incidence rate(%)** | **OR** | ***P*** | **Incidence rate(%)** | **OR** | ***P*** |  |
| **Chinese Criteria** |  |  |  |  |  |  | 0.222 |
| Chinese Criteria | 359 (32.5) | 1(Ref) |  | 624 (35.1) | 1(Ref) |  |  |
| WHO general Criteria | 324 (37.7) | 1.12 (0.92~1.35) | 0.27 | 485 (38.5) | 1.08 (0.92~1.26) | 0.343 |  |
| WHO Asia Pacific Criteria | 102 (44.3) | 1.43 (1.06~1.94) | 0.019 | 170 (39) | 1.07 (0.86~1.34) | 0.527 |  |
| **WHO general Criteria** |  |  |  |  |  |  | 0.183 |
| Chinese Criteria | 467 (33.1) | 1(Ref) |  | 776 (35.4) | 1(Ref) |  |  |
| WHO general Criteria | 276 (39.8) | 1.22 (1~1.48) | 0.046 | 435 (39.3) | 1.1 (0.94~1.28) | 0.235 |  |
| WHO Asia Pacific Criteria | 42 (47.7) | 1.56 (1~2.44) | 0.048 | 68 (38.2) | 1.02 (0.74~1.41) | 0.898 |  |
| **WHO Asia Pacific Criteria** |  |  |  |  |  |  | 0.308 |
| Chinese Criteria | 250 (31.4) | 1(Ref) |  | 483 (35.3) | 1(Ref) |  |  |
| WHO general Criteria | 217 (35.2) | 1.05 (0.84~1.33) | 0.653 | 293 (35.8) | 0.99 (0.82~1.19) | 0.905 |  |
| WHO Asia Pacific Criteria | 318 (40.7) | 1.28 (1.03~1.6) | 0.024 | 503 (39.1) | 1.08 (0.92~1.27) | 0.344 |  |

Adjust for gender, age, marriage status, smoking status, alcohol consumption, allergy history, and waist circumference.

**Table S2(d) Subgroup Analysis by Gender: Association Between Different BMI Criteria and Hyperuricemia.**

| **Hyperuricemia** | **Male** | | | **Female** | | | **P for interaction** |
| --- | --- | --- | --- | --- | --- | --- | --- |
|  | **Incidence rate(%)** | **OR** | ***P*** | **Incidence rate(%)** | **OR** | ***P*** |  |
| **Chinese Criteria** |  |  |  |  |  |  | 0.06 |
| Chinese Criteria | 456 (42.9) | 1(Ref) |  | 359 (21.2) | 1(Ref) |  |  |
| WHO general Criteria | 462 (56.5) | 1.73 (1.43~2.09) | <0.001 | 415 (34.6) | 1.73 (1.42~2.09) | <0.001 |  |
| WHO Asia Pacific Criteria | 128 (58.2) | 1.78 (1.32~2.41) | <0.001 | 185 (43.3) | 2.11 (1.59~2.81) | <0.001 |  |
| **WHO general Criteria** |  |  |  |  |  |  | 0.098 |
| Chinese Criteria | 608 (44.9) | 1(Ref) |  | 477 (22.8) | 1(Ref) |  |  |
| WHO general Criteria | 391 (58.9) | 1.71 (1.41~2.07) | <0.001 | 407 (38.5) | 1.7 (1.4~2.05) | <0.001 |  |
| WHO Asia Pacific Criteria | 47 (56) | 1.49 (0.95~2.34) | 0.079 | 75 (43.1) | 1.63 (1.12~2.37) | 0.011 |  |
| **WHO Asia Pacific Criteria** |  |  |  |  |  |  | 0.13 |
| Chinese Criteria | 315 (41.1) | 1(Ref) |  | 255 (19.4) | 1(Ref) |  |  |
| WHO general Criteria | 293 (49.9) | 1.46 (1.17~1.83) | 0.001 | 222 (28.5) | 1.55 (1.24~1.93) | <0.001 |  |
| WHO Asia Pacific Criteria | 438 (58.6) | 2 (1.62~2.48) | <0.001 | 482 (39.2) | 2.16 (1.72~2.71) | <0.001 |  |

Adjust for gender, age, marriage status, smoking status, alcohol consumption, allergy history, and waist circumference.

**Table S2(e) Subgroup Analysis by Gender: Association Between Different BMI Criteria and Hyperhomocysteinemia.**

| **Hyperhomocysteinemia** | **Male** | | | **Female** | | | **P for interaction** |
| --- | --- | --- | --- | --- | --- | --- | --- |
|  | **Incidence rate(%)** | **OR** | ***P*** | **Incidence rate(%)** | **OR** | ***P*** |  |
| **Chinese Criteria** |  |  |  |  |  |  | 0.28 |
| Chinese Criteria | 937 (93.8) | 1(Ref) |  | 1368 (85.7) | 1(Ref) |  |  |
| WHO general Criteria | 784 (96.8) | 2.06 (1.28~3.32) | 0.003 | 1051 (88.8) | 1.45 (1.14~1.84) | 0.002 |  |
| WHO Asia Pacific Criteria | 210 (96.8) | 2.54 (1.11~5.79) | 0.027 | 380 (90.5) | 1.85 (1.27~2.7) | 0.001 |  |
| **WHO general Criteria** |  |  |  |  |  |  | 0.96 |
| Chinese Criteria | 1215 (94.5) | 1(Ref) |  | 1694 (85.5) | 1(Ref) |  |  |
| WHO general Criteria | 638 (97) | 1.99 (1.18~3.35) | 0.01 | 953 (91.1) | 1.9 (1.47~2.46) | <0.001 |  |
| WHO Asia Pacific Criteria | 78 (95.1) | 1.53 (0.52~4.49) | 0.44 | 152 (88.9) | 1.57 (0.94~2.63) | 0.087 |  |
| **WHO Asia Pacific Criteria** |  |  |  |  |  |  | 0.036 |
| Chinese Criteria | 664 (93.1) | 1(Ref) |  | 1063 (85.9) | 1(Ref) |  |  |
| WHO general Criteria | 551 (96.2) | 1.97 (1.17~3.33) | 0.011 | 631 (84.8) | 0.97 (0.75~1.27) | 0.84 |  |
| WHO Asia Pacific Criteria | 716 (96.8) | 2.54 (1.52~4.25) | <0.001 | 1105 (90.8) | 1.83 (1.4~2.38) | <0.001 |  |

Adjust for gender, age, marriage status, smoking status, alcohol consumption, allergy history, and waist circumference.

**Table S2(f) Subgroup Analysis by Gender: Association Between Different BMI Criteria and Multiple metabolic risk(≥2) .**

| **Multiple metabolic risk**  **≥2** | **Male** | | | **Female** | | | **P for interaction** |
| --- | --- | --- | --- | --- | --- | --- | --- |
|  | **Incidence rate(%)** | **OR** | ***P*** | **Incidence rate(%)** | **OR** | ***P*** |  |
| **Chinese Criteria** |  |  |  |  |  |  | 0.05 |
| Chinese Criteria | 914 (82.8) | 1(Ref) |  | 1445 (81.3) | 1(Ref) |  |  |
| WHO general Criteria | 782 (91) | 1.83 (1.32~2.53) | <0.001 | 1108 (87.9) | 1.31 (1.03~1.68) | 0.031 |  |
| WHO Asia Pacific Criteria | 218 (94.8) | 3.35 (1.72~6.53) | <0.001 | 389 (89.2) | 1.3 (0.87~1.95) | 0.192 |  |
| **WHO general Criteria** |  |  |  |  |  |  | 0.09 |
| Chinese Criteria | 1188 (84.1) | 1(Ref) |  | 1798 (82.1) | 1(Ref) |  |  |
| WHO general Criteria | 644 (92.9) | 2.03 (1.42~2.91) | <0.001 | 987 (89.2) | 1.29 (0.99~1.67) | 0.059 |  |
| WHO Asia Pacific Criteria | 82 (93.2) | 2.2 (0.88~5.46) | 0.09 | 157 (88.2) | 0.95 (0.55~1.62) | 0.841 |  |
| **WHO Asia Pacific Criteria** |  |  |  |  |  |  | 0.057 |
| Chinese Criteria | 647 (81.4) | 1(Ref) |  | 1112 (81.2) | 1(Ref) |  |  |
| WHO general Criteria | 541 (87.7) | 1.52 (1.08~2.13) | 0.016 | 686 (83.8) | 1.16 (0.89~1.52) | 0.267 |  |
| WHO Asia Pacific Criteria | 726 (93) | 2.61 (1.75~3.9) | <0.001 | 1144 (89) | 1.36 (1.02~1.82) | 0.039 |  |

Adjust for gender, age, marriage status, smoking status, alcohol consumption, allergy history, and waist circumference.

**Table S2(g) Subgroup Analysis by Gender: Association Between Different BMI Criteria and Multiple metabolic risk(≥3).**

| **Multiple metabolic risk**  **(≥3)** | **Male** | | | **Female** | | | **P for interaction** |
| --- | --- | --- | --- | --- | --- | --- | --- |
|  | **Incidence rate(%)** | **OR** | ***P*** | **Incidence rate(%)** | **OR** | ***P*** |  |
| **Chinese Criteria** |  |  |  |  |  |  | 0.58 |
| Chinese Criteria | 620 (56.2) | 1(Ref) |  | 910 (51.2) | 1(Ref) |  |  |
| WHO general Criteria | 617 (71.8) | 1.64 (1.32~2.04) | <0.001 | 823 (65.3) | 1.43 (1.2~1.7) | <0.001 |  |
| WHO Asia Pacific Criteria | 181 (78.7) | 2.07 (1.4~3.07) | <0.001 | 310 (71.1) | 1.58 (1.2~2.09) | 0.001 |  |
| **WHO general Criteria** |  |  |  |  |  |  | 0.286 |
| Chinese Criteria | 827 (58.6) | 1(Ref) |  | 1167 (53.3) | 1(Ref) |  |  |
| WHO general Criteria | 522 (75.3) | 1.74 (1.38~2.19) | <0.001 | 755 (68.2) | 1.36 (1.13~1.63) | 0.001 |  |
| WHO Asia Pacific Criteria | 69 (78.4) | 1.76 (0.99~3.11) | 0.052 | 121 (68) | 1.05 (0.72~1.53) | 0.784 |  |
| **WHO Asia Pacific Criteria** |  |  |  |  |  |  | 0.066 |
| Chinese Criteria | 410 (51.6) | 1(Ref) |  | 685 (50) | 1(Ref) |  |  |
| WHO general Criteria | 417 (67.6) | 1.77 (1.39~2.25) | <0.001 | 482 (58.9) | 1.3 (1.07~1.57) | 0.008 |  |
| WHO Asia Pacific Criteria | 591 (75.7) | 2.45 (1.88~3.2) | <0.001 | 876 (68.2) | 1.54 (1.25~1.89) | <0.001 |  |

Adjust for gender, age, marriage status, smoking status, alcohol consumption, allergy history, and waist circumference.

**Table S2(h) Subgroup Analysis by Gender: Association Between Different BMI Criteria and Multiple metabolic risk(≥4).**

| **Multiple metabolic risk**  **(≥4)** | **Male** | | | **Female** | | | **P for interaction** |
| --- | --- | --- | --- | --- | --- | --- | --- |
|  | **Incidence rate(%)** | **OR** | ***P*** | **Incidence rate(%)** | **OR** | ***P*** |  |
| **Chinese Criteria** |  |  |  |  |  |  | 0.61 |
| Chinese Criteria | 274 (24.8) | 1(Ref) |  | 345 (19.4) | 1(Ref) |  |  |
| WHO general Criteria | 310 (36.1) | 1.6 (1.31~1.95) | <0.001 | 365 (28.9) | 1.64 (1.38~1.94) | <0.001 |  |
| WHO Asia Pacific Criteria | 109 (47.4) | 2.48 (1.84~3.34) | <0.001 | 154 (35.3) | 2.16 (1.71~2.73) | <0.001 |  |
| **WHO general Criteria** |  |  |  |  |  |  | 0.333 |
| Chinese Criteria | 363 (25.7) | 1(Ref) |  | 448 (20.5) | 1(Ref) |  |  |
| WHO general Criteria | 286 (41.3) | 1.92 (1.58~2.34) | <0.001 | 355 (32.1) | 1.76 (1.49~2.08) | <0.001 |  |
| WHO Asia Pacific Criteria | 44 (50) | 2.68 (1.73~4.17) | <0.001 | 61 (34.3) | 1.92 (1.38~2.67) | <0.001 |  |
| **WHO Asia Pacific Criteria** |  |  |  |  |  |  | 0.403 |
| Chinese Criteria | 174 (21.9) | 1(Ref) |  | 252 (18.4) | 1(Ref) |  |  |
| WHO general Criteria | 189 (30.6) | 1.46 (1.15~1.87) | 0.002 | 196 (23.9) | 1.37 (1.11~1.7) | 0.004 |  |
| WHO Asia Pacific Criteria | 330 (42.3) | 2.39 (1.91~3) | <0.001 | 416 (32.4) | 2.03 (1.69~2.43) | <0.001 |  |

Adjust for gender, age, marriage status, smoking status, alcohol consumption, allergy history, and waist circumference.

**Table S2(i) Subgroup Analysis by Gender: Association Between Different BMI Criteria and Multiple metabolic risk(5).**

| **Multiple metabolic risk**  **(5)** | **Male** | | | **Female** | | | **P for interaction** |
| --- | --- | --- | --- | --- | --- | --- | --- |
|  | **Incidence rate(%)** | **OR** | ***P*** | **Incidence rate(%)** | **OR** | ***P*** |  |
| **Chinese Criteria** |  |  |  |  |  |  | 0.555 |
| Chinese Criteria | 52 (4.7) | 1(Ref) |  | 62 (3.5) | 1(Ref) |  |  |
| WHO general Criteria | 71 (8.3) | 1.68 (1.15~2.44) | 0.007 | 84 (6.7) | 1.87 (1.33~2.63) | <0.001 |  |
| WHO Asia Pacific Criteria | 29 (12.6) | 2.72 (1.66~4.46) | <0.001 | 33 (7.6) | 2.2 (1.41~3.41) | <0.001 |  |
| **WHO general Criteria** |  |  |  |  |  |  | 0.103 |
| Chinese Criteria | 79 (5.6) | 1(Ref) |  | 85 (3.9) | 1(Ref) |  |  |
| WHO general Criteria | 60 (8.7) | 1.52 (1.07~2.17) | 0.02 | 85 (7.7) | 1.94 (1.42~2.66) | <0.001 |  |
| WHO Asia Pacific Criteria | 13 (14.8) | 2.69 (1.41~5.13) | 0.003 | 9 (5.1) | 1.29 (0.63~2.61) | 0.487 |  |
| **WHO Asia Pacific Criteria** |  |  |  |  |  |  | 0.957 |
| Chinese Criteria | 34 (4.3) | 1(Ref) |  | 43 (3.1) | 1(Ref) |  |  |
| WHO general Criteria | 45 (7.3) | 1.61 (1.01~2.56) | 0.046 | 42 (5.1) | 1.62 (1.05~2.51) | 0.029 |  |
| WHO Asia Pacific Criteria | 73 (9.3) | 2.09 (1.37~3.21) | 0.001 | 94 (7.3) | 2.29 (1.57~3.32) | <0.001 |  |

Adjust for gender, age, marriage status, smoking status, alcohol consumption, allergy history, and waist circumference.
