# Supplementary material for: Comparative analysis of three BMI cutoffs for five metabolic abnormalities in the population of Western Guangdong, China
Source: Front Nutr. 2025 Dec 3;12:1732345. doi: 10.3389/fnut.2025.1732345 (PMC12708589; doi:10.3389/fnut.2025.1732345)
Supplement: Supplementary file 3 [file Table_3.docx]

**Table S3(a) Subgroup Analysis by Gender: Association Between Different BMI Criteria and Hypertension.**

| **Hypetension** | **Age<69** | | | **Age≥69** | | | **P for interaction** |
| --- | --- | --- | --- | --- | --- | --- | --- |
|  | **Incidence rate(%)** | **OR** | ***P*** | **Incidence**  **rate(%)** | **OR** | ***P*** |  |
| **Chinese Criteria** |  |  |  |  |  |  | 0.06 |
| Chinese Criteria | 632 (53) | 1(Ref) |  | 990 (58.6) | 1(Ref) |  |  |
| WHO general Criteria | 658 (65.4) | 1.61 (1.35~1.92) | <0.001 | 770 (69.1) | 1.47 (1.24~1.73) | <0.001 |  |
| WHO Asia Pacific Criteria | 260 (74.7) | 2.62 (1.99~3.44) | <0.001 | 231 (72.6) | 1.63 (1.24~2.15) | <0.001 |  |
| **WHO general Criteria** |  |  |  |  |  |  |  |
| Chinese Criteria | 862 (55.7) | 1(Ref) |  | 1224 (59.6) | 1(Ref) |  | 0.55 |
| WHO general Criteria | 577 (67.8) | 1.63 (1.36~1.95) | <0.001 | 679 (71.5) | 1.55 (1.3~1.84) | <0.001 |  |
| WHO Asia Pacific Criteria | 111 (75) | 2.45 (1.66~3.63) | <0.001 | 88 (74.6) | 1.73 (1.13~2.67) | 0.012 |  |
| **WHO Asia Pacific Criteria** |  |  |  |  |  |  |  |
| Chinese Criteria | 447 (50.6) | 1(Ref) |  | 726 (56.7) | 1(Ref) |  | 0.493 |
| WHO general Criteria | 415 (62.5) | 1.56 (1.26~1.93) | <0.001 | 498 (64.5) | 1.43 (1.17~1.73) | <0.001 |  |
| WHO Asia Pacific Criteria | 688 (68.9) | 2.09 (1.72~2.53) | <0.001 | 767 (71.9) | 1.8 (1.5~2.15) | <0.001 |  |

Adjust for gender, age, marriage status, smoking status, alcohol consumption, allergy history, and waist circumference.

**Table S3(b) Subgroup Analysis by Gender: Association Between Different BMI Criteria and Dyslipidemia**

| **Dyslipidemia** | **Age<69** | | | **Age≥69** | | | **P for interaction** |
| --- | --- | --- | --- | --- | --- | --- | --- |
|  | **Incidence rate(%)** | **OR** | ***P*** | **Incidence**  **rate(%)** | **OR** | ***P*** |  |
| **Chinese Criteria** |  |  |  |  |  |  | 0.6 |
| Chinese Criteria | 725 (60.8) | 1(Ref) |  | 983 (58.2) | 1(Ref) |  |  |
| WHO general Criteria | 638 (63.4) | 1.12 (0.94~1.34) | 0.218 | 682 (61.2) | 1.14 (0.97~1.34) | 0.103 |  |
| WHO Asia Pacific Criteria | 230 (66.1) | 1.21 (0.93~1.57) | 0.152 | 190 (59.7) | 1.06 (0.82~1.36) | 0.658 |  |
| **WHO general Criteria** |  |  |  |  |  |  | 0.282 |
| Chinese Criteria | 948 (61.2) | 1(Ref) |  | 1210 (58.9) | 1(Ref) |  |  |
| WHO general Criteria | 548 (64.4) | 1.12 (0.94~1.34) | 0.216 | 582 (61.3) | 1.12 (0.95~1.31) | 0.182 |  |
| WHO Asia Pacific Criteria | 97 (65.5) | 1.13 (0.79~1.63) | 0.498 | 63 (53.4) | 0.78 (0.54~1.14) | 0.203 |  |
| **WHO Asia Pacific Criteria** |  |  |  |  |  |  | 0.752 |
| Chinese Criteria | 524 (59.3) | 1(Ref) |  | 736 (57.5) | 1(Ref) |  |  |
| WHO general Criteria | 424 (63.9) | 1.22 (0.98~1.51) | 0.074 | 474 (61.4) | 1.22 (1.01~1.47) | 0.039 |  |
| WHO Asia Pacific Criteria | 645 (64.6) | 1.23 (1.01~1.49) | 0.042 | 645 (60.4) | 1.16 (0.98~1.37) | 0.095 |  |

Adjust for gender, age, marriage status, smoking status, alcohol consumption, allergy history, and waist circumference.

**Table S3(c) Subgroup Analysis by Gender: Association Between Different BMI Criteria and Diabetes.**

| **Diabetes** | **Age<69** | | | **Age≥69** | | | **P for interaction** |
| --- | --- | --- | --- | --- | --- | --- | --- |
|  | **Incidence rate(%)** | **OR** | ***P*** | **Incidence**  **rate(%)** | **OR** | ***P*** |  |
| **Chinese Criteria** |  |  |  |  |  |  | 0.276 |
| Chinese Criteria | 445 (37.3) | 1(Ref) |  | 538 (31.9) | 1(Ref) |  |  |
| WHO general Criteria | 403 (40.1) | 1.05 (0.88~1.25) | 0.61 | 406 (36.4) | 1.15 (0.98~1.36) | 0.094 |  |
| WHO Asia Pacific Criteria | 139 (39.9) | 1.05 (0.82~1.36) | 0.687 | 133 (41.8) | 1.41 (1.1~1.82) | 0.007 |  |
| **WHO general Criteria** |  |  |  |  |  |  | 0.291 |
| Chinese Criteria | 580 (37.5) | 1(Ref) |  | 663 (32.3) | 1(Ref) |  |  |
| WHO general Criteria | 348 (40.9) | 1.09 (0.91~1.3) | 0.342 | 363 (38.3) | 1.22 (1.04~1.44) | 0.016 |  |
| WHO Asia Pacific Criteria | 59 (39.9) | 1.02 (0.71~1.45) | 0.927 | 51 (43.2) | 1.5 (1.02~2.19) | 0.038 |  |
| **WHO Asia Pacific Criteria** |  |  |  |  |  |  | 0.294 |
| Chinese Criteria | 332 (37.6) | 1(Ref) |  | 401 (31.3) | 1(Ref) |  |  |
| WHO general Criteria | 248 (37.3) | 0.92 (0.74~1.14) | 0.465 | 262 (33.9) | 1.1 (0.9~1.34) | 0.343 |  |
| WHO Asia Pacific Criteria | 407 (40.7) | 1.04 (0.86~1.26) | 0.687 | 414 (38.8) | 1.3 (1.09~1.55) | 0.004 |  |

Adjust for gender, age, marriage status, smoking status, alcohol consumption, allergy history, and waist circumference.

**Table S3(d) Subgroup Analysis by Gender: Association Between Different BMI Criteria and Hyperuricemia**

| **Hyperuricemia** | **Age<69** | | | **Age≥69** | | | **P for interaction** |
| --- | --- | --- | --- | --- | --- | --- | --- |
|  | **Incidence rate(%)** | **OR** | ***P*** | **Incidence**  **rate(%)** | **OR** | ***P*** |  |
| **Chinese Criteria** |  |  |  |  |  |  | 0.426 |
| Chinese Criteria | 309 (28.2) | 1(Ref) |  | 506 (30.4) | 1(Ref) |  |  |
| WHO general Criteria | 398 (43.6) | 2 (1.65~2.42) | <0.001 | 479 (43.3) | 1.57 (1.3~1.9) | <0.001 |  |
| WHO Asia Pacific Criteria | 153 (46.2) | 2.31 (1.77~3.01) | <0.001 | 160 (50.6) | 2.04 (1.51~2.77) | <0.001 |  |
| **WHO general Criteria** |  |  |  |  |  |  | 0.669 |
| Chinese Criteria | 446 (31.4) | 1(Ref) |  | 639 (31.5) | 1(Ref) |  |  |
| WHO general Criteria | 350 (44.9) | 1.83 (1.52~2.21) | <0.001 | 448 (47.6) | 1.73 (1.43~2.09) | <0.001 |  |
| WHO Asia Pacific Criteria | 64 (45.7) | 1.97 (1.37~2.83) | <0.001 | 58 (49.2) | 1.68 (1.1~2.58) | 0.016 |  |
| **WHO Asia Pacific Criteria** |  |  |  |  |  |  | 0.08 |
| Chinese Criteria | 202 (24.8) | 1(Ref) |  | 368 (29.1) | 1(Ref) |  |  |
| WHO general Criteria | 244 (40.3) | 1.94 (1.53~2.46) | <0.001 | 271 (35.7) | 1.25 (1.01~1.55) | 0.039 |  |
| WHO Asia Pacific Criteria | 414 (45) | 2.53 (2.04~3.13) | <0.001 | 506 (47.7) | 1.95 (1.56~2.44) | <0.001 |  |

Adjust for gender, age, marriage status, smoking status, alcohol consumption, allergy history, and waist circumference.

**Table S3(e) Subgroup Analysis by Gender: Association Between Different BMI Criteria and Hyperhomocysteinemia.**

| **Hyperhomocysteinemia** | **Age<69** | | | **Age≥69** | | | **P for interaction** |
| --- | --- | --- | --- | --- | --- | --- | --- |
|  | **Incidence rate(%)** | **OR** | ***P*** | **Incidence**  **rate(%)** | **OR** | ***P*** |  |
| **Chinese Criteria** |  |  |  |  |  |  | 0.193 |
| Chinese Criteria | 881 (84.4) | 1(Ref) |  | 1424 (91.8) | 1(Ref) |  |  |
| WHO general Criteria | 796 (87.8) | 1.29 (0.99~1.68) | 0.064 | 1039 (95.7) | 1.91 (1.33~2.75) | <0.001 |  |
| WHO Asia Pacific Criteria | 290 (89.5) | 1.58 (1.05~2.38) | 0.027 | 300 (95.8) | 2.07 (1.11~3.86) | 0.022 |  |
| **WHO general Criteria** |  |  |  |  |  |  |  |
| Chinese Criteria | 1153 (84.5) | 1(Ref) |  | 1756 (92.2) | 1(Ref) |  | 0.251 |
| WHO general Criteria | 693 (89.5) | 1.58 (1.19~2.09) | 0.002 | 898 (96.6) | 2.3 (1.5~3.51) | <0.001 |  |
| WHO Asia Pacific Criteria | 121 (88.3) | 1.42 (0.82~2.48) | 0.214 | 109 (94) | 1.33 (0.58~3.07) | 0.504 |  |
| **WHO Asia Pacific Criteria** |  |  |  |  |  |  |  |
| Chinese Criteria | 649 (84.3) | 1(Ref) |  | 1078 (91.3) | 1(Ref) |  | 0.087 |
| WHO general Criteria | 504 (84.8) | 0.91 (0.67~1.24) | 0.545 | 678 (93.8) | 1.37 (0.95~1.98) | 0.095 |  |
| WHO Asia Pacific Criteria | 814 (89.4) | 1.49 (1.1~2.01) | 0.009 | 1007 (96.3) | 2.43 (1.65~3.58) | <0.001 |  |

Adjust for gender, age, marriage status, smoking status, alcohol consumption, allergy history, and waist circumference.

**Table S3(f) Subgroup Analysis by Gender: Association Between Different BMI Criteria and Multiple metabolic risk(≥2) .**

| **Multiple metabolic risk**  **≥2** | **Age<69** | | | **Age≥69** | | | **P for interaction** |
| --- | --- | --- | --- | --- | --- | --- | --- |
|  | **Incidence rate(%)** | **OR** | ***P*** | **Incidence rate(%)** | **OR** | ***P*** |  |
| **Chinese Criteria** |  |  |  |  |  |  | 0.356 |
| Chinese Criteria | 936 (78.5) | 1(Ref) |  | 1423 (84.3) | 1(Ref) |  |  |
| WHO general Criteria | 858 (85.3) | 1.46 (1.15~1.84) | 0.002 | 1032 (92.6) | 1.34 (0.98~1.83) | 0.068 |  |
| WHO Asia Pacific Criteria | 310 (89.1) | 2 (1.37~2.91) | <0.001 | 297 (93.4) | 1.08 (0.63~1.85) | 0.776 |  |
| **WHO general Criteria** |  |  |  |  |  |  | 0.213 |
| Chinese Criteria | 1235 (79.8) | 1(Ref) |  | 688 (77.8) | 1(Ref) |  |  |
| WHO general Criteria | 738 (86.7) | 1.42 (1.01~2) | 0.043 | 547 (82.4) | 1.22 (0.93~1.61) | 0.043 |  |
| WHO Asia Pacific Criteria | 131 (88.5) | 0.67 (0.33~1.36) | 0.268 | 869 (87) | 1.68 (1.3~2.19) | 0.268 |  |
| **WHO Asia Pacific Criteria** |  |  |  |  |  |  | 0.286 |
| Chinese Criteria | 688 (77.8) | 1(Ref) |  | 1071 (83.6) | 1(Ref) |  |  |
| WHO general Criteria | 547 (82.4) | 1.22 (0.93~1.61) | 0.142 | 680 (88.1) | 1.15 (0.84~1.59) | 0.384 |  |
| WHO Asia Pacific Criteria | 869 (87) | 1.68 (1.3~2.19) | <0.001 | 1001 (93.8) | 1.42 (0.99~2.03) | 0.056 |  |

Adjust for gender, age, marriage status, smoking status, alcohol consumption, allergy history, and waist circumference.

**Table S3(g) Subgroup Analysis by Gender: Association Between Different BMI Criteria and Multiple metabolic risk(≥3).**

| **Multiple metabolic risk**  **≥3** | **Age<69** | | | **Age≥69** | | | **P for interaction** |
| --- | --- | --- | --- | --- | --- | --- | --- |
|  | **Incidence rate(%)** | **OR** | ***P*** | **Incidence rate(%)** | **OR** | ***P*** |  |
| **Chinese Criteria** |  |  |  |  |  |  | 0.24 |
| Chinese Criteria | 608 (51) | 1(Ref) |  | 922 (54.6) | 1(Ref) |  |  |
| WHO general Criteria | 647 (64.3) | 1.6 (1.34~1.92) | <0.001 | 793 (71.2) | 1.36 (1.12~1.65) | 0.002 |  |
| WHO Asia Pacific Criteria | 256 (73.6) | 2.43 (1.83~3.23) | <0.001 | 235 (73.9) | 1.16 (0.84~1.6) | 0.377 |  |
| **WHO general Criteria** |  |  |  |  |  |  | 0.603 |
| Chinese Criteria | 832 (53.7) | 1(Ref) |  | 1162 (56.6) | 1(Ref) |  |  |
| WHO general Criteria | 575 (67.6) | 1.61 (1.32~1.97) | <0.001 | 702 (74) | 1.38 (1.13~1.69) | 0.002 |  |
| WHO Asia Pacific Criteria | 104 (70.3) | 1.72 (1.13~2.61) | 0.011 | 86 (72.9) | 0.95 (0.6~1.5) | 0.811 |  |
| **WHO Asia Pacific Criteria** |  |  |  |  |  |  | 0.781 |
| Chinese Criteria | 425 (48.1) | 1(Ref) |  | 670 (52.3) | 1(Ref) |  |  |
| WHO general Criteria | 407 (61.3) | 1.53 (1.24~1.9) | <0.001 | 492 (63.7) | 1.33 (1.08~1.63) | 0.007 |  |
| WHO Asia Pacific Criteria | 679 (68) | 2.02 (1.64~2.49) | <0.001 | 788 (73.9) | 1.58 (1.26~1.98) | <0.001 |  |

Adjust for gender, age, marriage status, smoking status, alcohol consumption, allergy history, and waist circumference.

**Table S3(h) Subgroup Analysis by Gender: Association Between Different BMI Criteria and Multiple metabolic risk(≥4).**

| **Multiple metabolic risk**  **≥4** | **Age<69** | | | **Age≥69** | | | **P for interaction** |
| --- | --- | --- | --- | --- | --- | --- | --- |
|  | **Incidence rate(%)** | **OR** | ***P*** | **Incidence rate(%)** | **OR** | ***P*** |  |
| **Chinese Criteria** |  |  |  |  |  |  | 0.648 |
| Chinese Criteria | 245 (20.5) | 1(Ref) |  | 374 (22.2) | 1(Ref) |  |  |
| WHO general Criteria | 316 (31.4) | 1.68 (1.38~2.04) | <0.001 | 359 (32.2) | 1.57 (1.32~1.86) | <0.001 |  |
| WHO Asia Pacific Criteria | 128 (36.8) | 2.17 (1.67~2.83) | <0.001 | 135 (42.5) | 2.41 (1.87~3.12) | <0.001 |  |
| **WHO general Criteria** |  |  |  |  |  |  | 0.853 |
| Chinese Criteria | 339 (21.9) | 1(Ref) |  | 472 (23) | 1(Ref) |  |  |
| WHO general Criteria | 295 (34.7) | 1.84 (1.52~2.22) | <0.001 | 346 (36.5) | 1.82 (1.53~2.16) | <0.001 |  |
| WHO Asia Pacific Criteria | 55 (37.2) | 2.08 (1.45~2.98) | <0.001 | 50 (42.4) | 2.37 (1.61~3.48) | <0.001 |  |
| **WHO Asia Pacific Criteria** |  |  |  |  |  |  | 0.923 |
| Chinese Criteria | 162 (18.3) | 1(Ref) |  | 264 (20.6) | 1(Ref) |  |  |
| WHO general Criteria | 177 (26.7) | 1.43 (1.11~1.83) | 0.005 | 208 (26.9) | 1.38 (1.12~1.71) | 0.003 |  |
| WHO Asia Pacific Criteria | 350 (35) | 2.21 (1.78~2.75) | <0.001 | 396 (37.1) | 2.13 (1.77~2.57) | <0.001 |  |

Adjust for gender, age, marriage status, smoking status, alcohol consumption, allergy history, and waist circumference.

**Table S3(i) Subgroup Analysis by Gender: Association Between Different BMI Criteria and Multiple metabolic risk(5)**

| **Multiple metabolic risk**  **5** | **Age<69** | | | **Age≥69** | | | **P for interaction** |
| --- | --- | --- | --- | --- | --- | --- | --- |
|  | **Incidence rate(%)** | **OR** | ***P*** | **Incidence rate(%)** | **OR** | ***P*** |  |
| **Chinese Criteria** |  |  |  |  |  |  | 0.968 |
| Chinese Criteria | 45 (3.8) | 1(Ref) |  | 69 (4.1) | 1(Ref) |  |  |
| WHO general Criteria | 73 (7.3) | 1.83 (1.25~2.69) | 0.002 | 82 (7.4) | 1.77 (1.27~2.47) | 0.001 |  |
| WHO Asia Pacific Criteria | 32 (9.2) | 2.47 (1.53~3.98) | <0.001 | 30 (9.4) | 2.35 (1.5~3.7) | <0.001 |  |
| **WHO general Criteria** |  |  |  |  |  |  | 0.486 |
| Chinese Criteria | 73 (4.7) | 1(Ref) |  | 91 (4.4) | 1(Ref) |  |  |
| WHO general Criteria | 63 (7.4) | 1.56 (1.1~2.22) | 0.013 | 82 (8.6) | 1.93 (1.41~2.64) | <0.001 |  |
| WHO Asia Pacific Criteria | 14 (9.5) | 2.11 (1.15~3.87) | 0.016 | 8 (6.8) | 1.53 (0.72~3.25) | 0.268 |  |
| **WHO Asia Pacific Criteria** |  |  |  |  |  |  | 0.342 |
| Chinese Criteria | 27 (3.1) | 1(Ref) |  | 50 (3.9) | 1(Ref) |  |  |
| WHO general Criteria | 46 (6.9) | 2.07 (1.27~3.4) | 0.004 | 41 (5.3) | 1.34 (0.88~2.06) | 0.173 |  |
| WHO Asia Pacific Criteria | 77 (7.7) | 2.42 (1.54~3.8) | <0.001 | 90 (8.4) | 2.13 (1.49~3.06) | <0.001 |  |

Adjust for gender, age, marriage status, smoking status, alcohol consumption, allergy history, and waist circumference.
